# Altered GABAergic signaling and chloride homeostasis in eye movement circuits during late neurodevelopment: implications for Alzheimer’s disease therapy

**DOI:** 10.3389/fphar.2025.1675799

**Published:** 2025-10-28

**Authors:** Christophe Porcher, Claudio Rivera, Igor Medina, Lejla Koric

**Affiliations:** ^1^ INMED, INSERM, Aix-Marseille University, Marseille, France; ^2^ Neuroscience Center, University of Helsinki, Helsinki, Finland; ^3^ Department of Neurology and Neuropsychology, CHU Timone, Assistance Publique-Hôpitaux de Marseille, Marseille, France; ^4^ Aix-Marseille University, UMR 7249, CNRS, Centrale Marseille, Institut Fresnel, Marseille, France

**Keywords:** Alzheimer, oculomotor, KCC2, GABA, chloride homeostasis

## Abstract

Eye movement deficits, including abnormal saccades and impaired smooth pursuits, are among the earliest observable indicators of neurodegenerative diseases, particularly Alzheimer’s disease (AD). These deficits arise from dysfunctions in neural circuits controlling oculomotor function, including the superior colliculus, parietal and frontal eye fields, cerebellum, and locus coeruleus (LC). Since these circuits rely on a delicate balance of excitation and inhibition (E/I), their impairment reflects broader neural dysregulation seen in neurodegenerative diseases. Notably, oculomotor abnormalities strongly correlate with cognitive decline and the progression of neuropathological hallmarks, highlighting their potential as sensitive, non-invasive clinical markers for early detection. GABAergic signaling, the principal mechanism of inhibitory neurotransmission, plays a central role in maintaining E/I balance and regulating neural network activity. In neurodegenerative diseases, GABAergic dysfunction is characterized by reduced GABA levels, altered GABA_A_ receptor function, and compromised inhibitory control. These changes drive network hyperexcitability, synaptic instability, and cognitive impairments. Such disruptions are particularly impactful in oculomotor circuits, contributing directly to eye movement deficits. The potassium-chloride co-transporter 2 (KCC2), a key regulator of intracellular chloride homeostasis, is essential for maintaining GABAergic inhibition. In AD, KCC2 dysfunction exacerbates GABAergic dysregulation, amplifying E/I imbalance and impairing neural circuits. This review integrates current findings on GABAergic signaling, KCC2 dysfunction, and oculomotor deficits in AD, offering novel insights into the mechanisms linking KCC2 dysfunction and oculomotor impairments within the context of AD.

## 1 Introduction

Alzheimer’s disease (AD) is a progressive neurodegenerative disorder characterized by the deterioration of interconnected brain networks essential for cognition. Growing evidence indicates that AD pathology selectively disrupts specific circuits, with amyloid and tau accumulation driving distinctive patterns of dysfunction (Buckner et al., 2005; Seeley et al., 2009; Jones, 2010; Sperling et al., 2010). Among the earliest affected are networks mediating visual attention and oculomotor control, which provide valuable insights into disease progression (Anderson and MacAskill, 2013; Wilcockson et al., 2019; Hannonen et al., 2022; Opwonya et al., 2023). Impairments in these systems compromise the brain’s capacity to filter, prioritize, and integrate sensory input, thereby accelerating decline in memory, executive function, and visuospatial processing (Rizzo et al., 2000; Quental et al., 2013; Eraslan Boz et al., 2024). Mechanistically, alterations in the excitatory/inhibitory (E/I) balance within vulnerable networks are emerging as key contributors to AD pathogenesis.

Here, we examine how early oculomotor anomalies, reflecting visual attention deficits in AD, provide critical insights into disease progression. We focus on GABAergic transmission dysfunction, a pivotal factor in the breakdown of inhibitory control within these circuits. The inhibitory action of GABA relies on the complementary functions of ionotropic GABA_A_​ receptors (GABA_A​_Rs) and metabotropic GABA_B_​ receptors (GABA_B_​Rs). GABA_A​_Rs are Cl^−^/HCO_3_^−^-permeable channels whose inhibitory efficacy depends on intracellular chloride concentration ([Cl^−^]ᵢ), which determines the reversal potential (E_GABA_). Under physiological conditions, low [Cl^−^]ᵢ maintains a negative E_GABA_​, such that GABA_A​_R activation produces hyperpolarizing or shunting inhibition. In AD, chloride homeostasis is disrupted, leading to depolarized E_GABA_​ and weakened inhibition (Chen et al., 2017). In extreme cases, depolarizing GABA_A​_Rs activation can trigger the opening of voltage-gated Ca^2+^ channels and facilitate NMDA receptor (NMDAR) activation (Leinekugel et al., 1997; Kilb, 2021). Notably, inhibition of NMDARs with memantine, a low-to-moderate affinity, uncompetitive, and voltage-dependent channel blocker, preferentially suppresses pathological overactivation while sparing physiological signaling, providing modest symptomatic benefit in moderate-to-severe AD (Kishi et al., 2017). In contrast, GABA_B_​Rs are GPCRs that mediate slow inhibition through activation of GIRK (Kir3) K^+^ channels and presynaptic Ca^2+^ channel inhibition, thereby maintaining membrane hyperpolarization via a chloride-independent pathway (Benarroch, 2012). Importantly, nanoscale remodeling of GABA_B_​R–GIRK complexes has been observed near amyloid plaques (Martín-Belmonte et al., 2022). Moreover, selective pharmacological activation of GIRK channels (e.g., ML297, VU0810464) restores hippocampal function and memory in AD models, identifying this pathway as a promising therapeutic strategy (Jeremic et al., 2021).

During early development, GABAergic signaling is predominantly excitatory due to chloride gradients regulated by transporters such as KCC2. As the nervous system matures, signaling shifts toward inhibition, establishing the excitatory/inhibitory (E/I) balance essential for network stability (Ben-Ari et al., 2007; Kaila et al., 2014). Disruptions in these late neurodevelopmental processes, particularly those involving KCC2-dependent maturation, may leave lasting circuit vulnerabilities that predispose to AD-related fragility. While oculomotor abnormalities can serve as early markers of altered E/I balance in pediatric populations, there is no direct evidence that they predict AD onset. For example, voluntary saccadic control depends on prefrontal E/I maturation; in children with neurodevelopmental challenges, deficits in this control signal atypical development but do not predict later neurodegeneration (Ibrahimi et al., 2024). Nonetheless, subtle oculomotor alterations may reveal persistent vulnerabilities shaped by disrupted GABAergic maturation. According to John Stein’s magnocellular theory, dyslexia arises from dysfunction in the magnocellular visual pathway, which is critical for motion processing and eye movement control (Stein, 2019). Individuals with dyslexia exhibit reduced visual motion sensitivity and binocular instability, leading to reading difficulties, perceptual instability, and letter reversals. Characteristic oculomotor features also include increased regressions, a higher number of fixations, and longer fixation durations (Bilbao et al., 2024). These neurobiological vulnerabilities underlie reading challenges in dyslexia and may predispose to degeneration of dorsal visual pathways later in life, potentially linking dyslexia with certain neurodegenerative conditions, such as primary progressive aphasia and the visual impairments seen in posterior cortical atrophy (PCA). Clinical heterogeneity in AD further supports this framework. Distinct AD phenotypes are thought to arise from selective vulnerabilities of specific brain networks (Mesulam et al., 2008; Rogalski et al., 2013; Miller et al., 2018), even when they share common hallmarks such as synaptic loss or amyloid deposition (Jones et al., 2016; Mahzarnia et al., 2023). Thus, AD manifestations emerge from the interplay between universal pathogenic processes and network-specific susceptibilities, which together shape symptom profiles and disease progression. These vulnerabilities may first appear as subtle oculomotor changes, remain compensated for years, and ultimately contribute to the cognitive decline characteristic of AD. Combined with genetic predispositions and cumulative lifespan risk factors, they may exacerbate E/I imbalance, potentially constituting a pre-amyloid mechanism that accelerates pathology. From this perspective, oculomotor alterations may represent intermediate phenotypes of neural E/I imbalance and thus warrant longitudinal study as candidate biomarkers for preclinical AD. By bridging the gap between subjective cognitive complaints and measurable network dysfunction, oculomotor features hold promises as clinically meaningful indicators. When integrated with genetic and neuroimaging data, they could substantially improve early risk identification. This review therefore adopts a late-neurodevelopmental perspective, emphasizing how altered GABAergic signaling and chloride regulation in eye-movement networks illuminate both shared and phenotype-specific mechanisms of AD. Integrating oculomotor assessments with molecular approaches aimed at restoring KCC2 function and GABAergic inhibitory tone may provide a comprehensive strategy that bridges neuroscience and clinical intervention in AD.

### 1.1 GABAergic signaling and chloride homeostasis in Alzheimer’s disease

The brain’s primary inhibitory neurotransmitter, GABA, ensures stable neural network dynamics and overall brain homeostasis (Ben-Ari, 2002). In AD, GABAergic signaling is disrupted, with reduced GABA levels, altered receptor function, and impaired inhibitory control contributing to cognitive decline, network instability, and an exacerbated E/I imbalance that accelerates disease progression (Zhou et al., 2021; Capsoni et al., 2022; Mao et al., 2024). While normal aging is associated with a decline in GABAergic signaling, including reduced glutamate decarboxylase (GAD) expression, altered interneuron subpopulations, and receptor subunit changes that impair plasticity (McQuail et al., 2015; Rozycka and Liguz-Lecznar, 2017), both postmortem and *in vivo* studies demonstrate that these deficits are more pronounced in AD, particularly within the hippocampus and prefrontal cortex (Rissman et al., 2007; Lanctôt et al., 2017; Jiménez-Balado and Eich, 2021; Zhou et al., 2021). Magnetic resonance spectroscopy further indicates that such reductions emerge as early as the mild cognitive impairment (MCI) stage (Mao et al., 2024). These deficits correlate with synaptic loss and neuronal degeneration, particularly in the entorhinal cortex and hippocampus, regions critical for memory, at levels exceeding those observed in age-matched controls (Xu et al., 2020; Jiménez-Balado and Eich, 2021; Scaduto et al., 2023). Crucially, AD pathology entails not only reduced GABA concentrations but also receptor dysfunction, altered subunit composition, and selective vulnerability of parvalbumin (PV) interneurons to Amyloid-β (Aβ) toxicity. The loss of PV interneurons, key regulators of gamma oscillations associated with memory and attention (Tessier et al., 2023), leads to network hyperactivity and disrupted oscillatory rhythms, thereby exacerbating cognitive impairment (Busche and Konnerth, 2016; Vericel et al., 2024; Wei et al., 2024). In parallel, both GABA_A_Rs and GABA_B_Rs functions are altered in AD. The density of GABA_A_Rs, particularly in the hippocampus and cortex, is reduced (Mao et al., 2024), and alterations in subunit composition, most notably affecting the α5 subunit, impair receptor function. Consistent with subunit-specific changes, inhibition of α5-containing GABA_A_Rs​ has been shown to enhance cognition by reducing tonic inhibition in hippocampal and prefrontal networks. By contrast, α1 expression is decreased, whereas β-subunits are relatively preserved in AD (Howell et al., 2000; Rissman et al., 2007), supporting the rationale for subunit-selective modulation combined with approaches that restore KCC2-dependent chloride homeostasis. Meanwhile, GABA_B_Rs dysfunction contributes to impaired neurotransmitter release and neuronal synchronization, further aggravating network instability and cognitive decline (Vico Varela et al., 2019).

**FIGURE 1:**
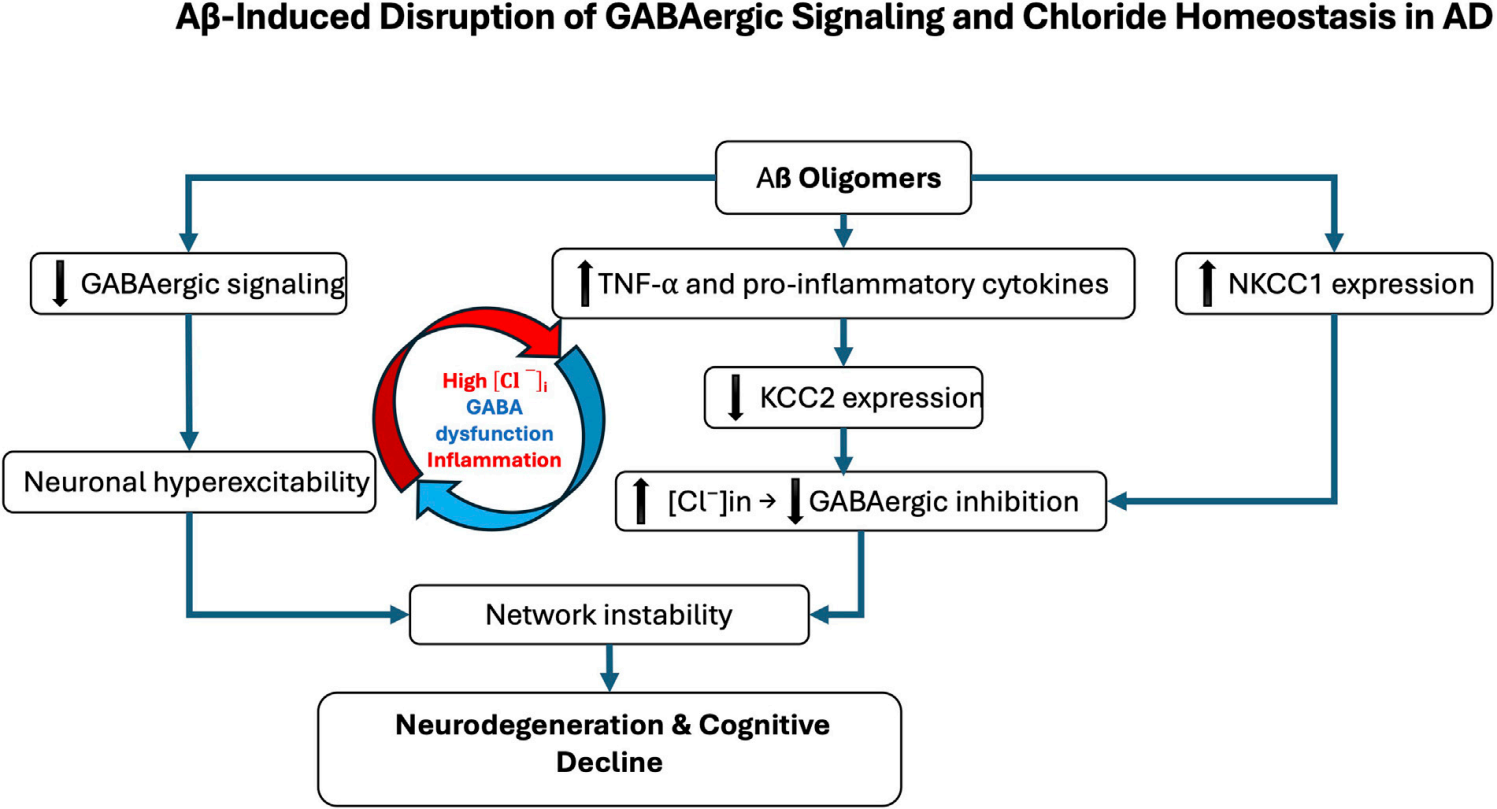
Aβ-induced disruption of GABAergic signaling and chloride homeostasis in Alzheimer’s disease (AD). Amyloid-β (Aβ) oligomers contribute to GABAergic dysfunction through multiple molecular mechanisms. They directly impair GABAergic transmission by inducing oxidative stress, altering synaptic function, and disrupting receptor trafficking. In parallel, Aβ promotes neuroinflammatory responses, notably through microglial activation and increased production of pro-inflammatory cytokines such as TNF-α. These inflammatory signals affect the expression of the potassium-chloride cotransporter KCC2, a key regulator of intracellular chloride concentration. In AD, KCC2 expression is downregulated, particularly in the hippocampus and prefrontal cortex, while the sodium-potassium-chloride cotransporter NKCC1 is aberrantly upregulated. This imbalance leads to intracellular chloride accumulation, weakening GABAergic inhibition. The resulting neuronal hyperexcitability contributes to neurodegeneration and the cognitive decline characteristic of AD. The red and blue arrows represent the vicious cycle linking Alzheimer’s disease, inflammatory processes, and GABAergic dysregulation.

At the molecular level, Aβ oligomers directly impair GABAergic signaling through oxidative stress, disruption of synaptic function, and interference with receptor trafficking (Zhou et al., 2021). The amyloid peptide β also promotes neuroinflammation, activating microglia and increasing pro-inflammatory cytokines like TNF-α, which modulate the expression of key proteins such as brain-derived neurotrophic factor (BDNF) and its receptor TrkB. This signaling cascade is crucial for the regulation of KCC2, which is essential for maintaining the inhibitory function of GABA_A_Rs (Heubl et al., 2017; Porcher et al., 2018; Hamze et al., 2024). Disruptions in chloride homeostasis are now recognized as a core mechanism of GABAergic dysfunction in AD. Under physiological conditions, KCC2 maintains low [Cl^−^]ᵢ, thereby allowing GABA to exert inhibitory effects (Medina et al., 2014). In AD, however, pathological alterations in the excitatory–inhibitory (E/I) balance have been documented, most notably the downregulation of KCC2 in hippocampal and prefrontal circuits, together with the aberrant upregulation of NKCC1, which is normally suppressed in mature neurons (Kreis et al., 2021; Lam et al., 2022; Del Turco et al., 2023; Lam et al., 2023; Barbour et al., 2024). This shift results in [Cl^−^]ᵢ accumulation, which impairs GABAergic inhibition, thereby promoting neuronal hyperexcitability and cognitive decline (Figure 1). Although inhibitory dysfunction predisposes neuronal networks to hyperexcitability in AD, several compensatory mechanisms initially stabilize excitatory activity and prevent uncontrolled firing. Even when GABAergic signaling becomes less hyperpolarizing, increased chloride conductance can still exert a shunting inhibitory effect that lowers membrane resistance and limits excitatory input efficacy (Buzsáki, 1984; Kaila et al., 2014). At the intrinsic level, neurons enhance potassium conductance via Kv7/KCNQ and GIRK channels, thereby stabilizing membrane potential and counteracting excessive depolarization (Sánchez-Rodríguez et al., 2019). In parallel, homeostatic synaptic plasticity adjusts excitatory drive through synaptic scaling and activity-dependent regulation of ion channel expression (Baculis et al., 2022; Wen and Turrigiano, 2024). Glial mechanisms are equally critical: astrocytes maintain ionic balance through Kir4.1-mediated K^+^ buffering and stabilize synapses by clearing glutamate via EAAT2 transporters, processes impaired in AD models and human cortex (Yeung et al., 2021; Wood et al., 2022; Kim et al., 2024; Samokhina et al., 2025; Srivastava et al., 2025). Neuromodulatory systems further provide tonic regulation of excitability: cholinergic inputs adjust firing dynamics through TRPM4-dependent mechanisms (Combe et al., 2023); dopaminergic projections modulate inhibitory control in prefrontal circuits (Di Domenico and Mapelli, 2023); serotonergic pathways exert receptor-specific actions, either enhancing inhibition (5-HT_1_A) or facilitating excitation (5-HT_2_A) (Celada et al., 2013; Salvan et al., 2023); and noradrenergic tone contributes to arousal-dependent gain control and E/I stabilization (Slater et al., 2022). Collectively, these mechanisms help delay overt network dysfunction in early AD. However, with disease progression, marked by interneuron loss, chloride transporter dysregulation, and astrocytic failure, these protective adaptations are ultimately overwhelmed, leading to network hyperexcitability, seizures, and cognitive decline.

Mechanistically, Aβ oligomers downregulate KCC2 by disrupting BDNF–TrkB signaling and impairing proBDNF maturation (Garzon and Fahnestock, 2007; Jerónimo-Santos et al., 2015; Zhou et al., 2021). Accumulated proBDNF preferentially activates p75^NTR^, promoting synaptic loss and destabilizing KCC2 through internalization, thereby amplifying chloride imbalance and shifting GABA action toward excitation (Riffault et al., 2014; 2016; Bie et al., 2022; Bruno et al., 2023; Hamze et al., 2024). These alterations create a vicious cycle in which chloride dysregulation, GABAergic failure, and neuroinflammation reinforce one another. Chronic inflammation exacerbates KCC2 downregulation, further destabilizing synapses and accelerating pathology (Tessier et al., 2023) (Figure 1). As a result, inhibition shifts from protective to maladaptive, contributing directly to network dysfunction and degeneration. Whether NKCC1/KCC2 alterations are primary drivers or secondary amplifiers remains unresolved. Signaling pathway through mTORC1, a key regulator of chloride transporters, is dysregulated early in AD, aggravating E/I imbalance and seizure susceptibility. Patients have a 6–10-fold higher seizure risk than age-matched controls (Pandis and Scarmeas, 2012; Xu et al., 2021; Zhang et al., 2022). Rapamycin, an mTORC1 inhibitor, restores NKCC1/KCC2 balance and reduces seizure pathology, whereas the contribution of the WNK/SPAK pathway to transporter regulation in AD remains largely unexplored (Barbour et al., 2024; Blum and Levi, 2024). Emerging evidence suggests that E/I imbalances established during neurodevelopment may act as permissive triggers of AD pathology. Altered interneuron function can precede amyloid deposition and clinical symptoms, driving early synaptic dysfunction and abnormal activity in hippocampal and prefrontal circuits (Xu et al., 2020; Lam et al., 2023). Soluble Aβ further impairs inhibition by targeting fast-spiking interneurons, promoting hyperexcitability that accelerates tau pathology and neuronal loss (Li et al., 2016; Ren et al., 2018). Subtype-specific features underscore this vulnerability: early-onset AD (EOAD) is characterized by denser tangles and more aggressive cortical pathology than late-onset AD (LOAD), likely accelerating transporter dysregulation and shifting GABAergic signaling toward excitation (van der Flier et al., 2011; Jagust, 2018). By contrast, in aging and LOAD, transporter alterations progress more gradually and may initially remain compensable (Schneider et al., 2007; Rahimi and Kovacs, 2014). Thus, while KCC2 downregulation and NKCC1 upregulation are consistent hallmarks of symptomatic AD, early developmental E/I disturbances may prime circuits for dysfunction. Clarifying the timing, regional specificity, and molecular regulation of chloride transporters across EOAD, LOAD, and aging will be critical to disentangle cause from consequence in disease trajectory (Jones et al., 2016; Mahzarnia et al., 2023).

### 1.2 KCC2 phosphorylation and its implications in Alzheimer’s disease

**FIGURE 2:**
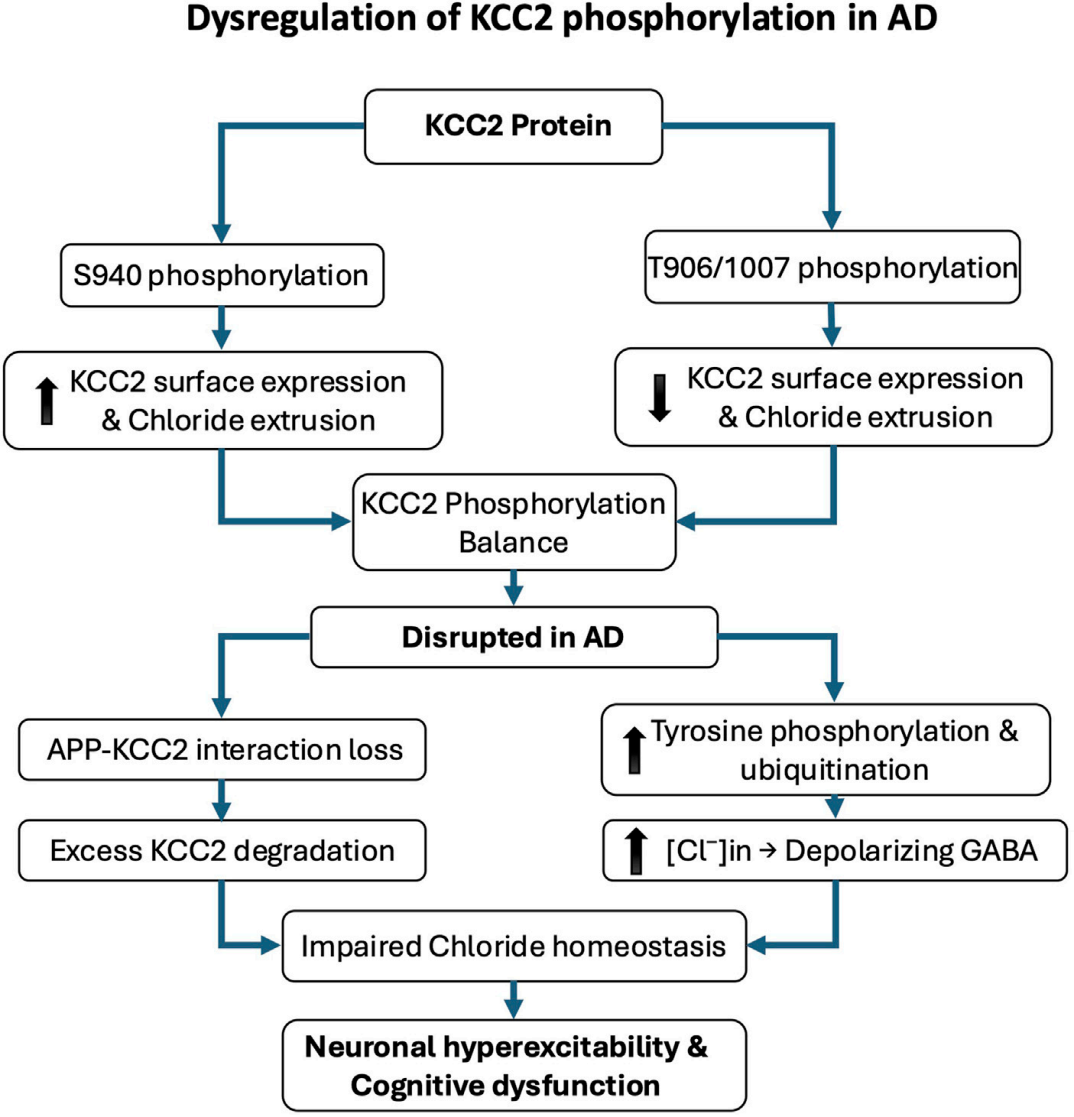
Dysregulation of KCC2 phosphorylation in Alzheimer’s disease (AD). KCC2 function is critically regulated by post-translational phosphorylation, which modulates its membrane expression and chloride efflux. Phosphorylation at serine 940 (S940) enhances KCC2 stability at the plasma membrane and promotes its activity, whereas phosphorylation at threonine residues 906 and 1007 (T906/1007) reduces surface expression and inhibits function. In AD, KCC2 activity is disrupted by imbalanced phosphorylation and increased degradation. The amyloid precursor protein (APP) normally limits KCC2 tyrosine phosphorylation; in its absence or dysfunction, KCC2 becomes hyperphosphorylated on tyrosine residues, leading to ubiquitination, degradation, and loss of function. These molecular alterations impair chloride homeostasis and shift GABAergic signaling toward excitation, contributing to neuronal hyperexcitability, cognitive decline, and early pathophysiological changes in AD.

Emerging research underscores a critical link between the phosphorylation state of KCC2 and the pathophysiology of AD. Alterations in KCC2 function have been shown to disrupt neuronal inhibition and contribute to cognitive decline (Keramidis et al., 2023). A major mechanism regulating KCC2 activity is post-translational modification, particularly phosphorylation. Phosphorylation at serine 940 (S940) enhances KCC2 membrane stability and functional expression, thereby promoting its activity. Conversely, phosphorylation at threonine residues 906 and 1007 inhibits KCC2 by reducing its surface expression and chloride transport capacity (Kahle et al., 2013; Kaila et al., 2014; Medina et al., 2014; Pethe et al., 2023). In AD, increasing evidence indicates that KCC2 function is impaired due to dysregulated phosphorylation and enhanced degradation. The amyloid precursor protein, a central player in AD pathology, has been shown to interact with KCC2 and limit its tyrosine phosphorylation. In the absence of amyloid precursor protein, KCC2 undergoes excessive tyrosine phosphorylation and ubiquitination, triggering its degradation and significantly reducing its expression and function (Chen et al., 2017). Such degradation leads to disrupted chloride homeostasis and depolarizing GABAergic responses, which in turn exacerbate neuronal hyperexcitability and cognitive dysfunction (Figure 2). Notably, AD mouse models, particularly those expressing amyloid precursor protein mutations, exhibit early impairments in KCC2 expression and function that precede overt amyloid deposition and synaptic degeneration. These early deficits are accompanied by increased levels of Aβ 42 and correlate with learning and memory impairments (Keramidis et al., 2023).

## 2 Eye movement deficits as a marker for neurodegeneration

### 2.1 Oculomotor system and neural circuits

Eye movements depend on the intricate coordination of complex neural networks that govern their initiation, execution, regulation, and precision (Massone, 1994). These movements are broadly categorized into extrinsic and intrinsic types. Extrinsic eye movements encompass a variety of oculomotor functions, including rapid saccades, steady fixations, convergent and divergent vergence, smooth pursuit of moving targets, and various oculomotor reflexes. These movements, primarily controlled by the extraocular muscles, play a crucial role in positioning and stabilizing the eyes. Intrinsic eye movements, on the other hand, are limited to pupillary adjustments that regulate light intake and contribute to visual acuity. This distinction underscores the complexity and adaptability of the oculomotor system in responding to both external visual stimuli and internal cognitive demands.

**FIGURE 3:**
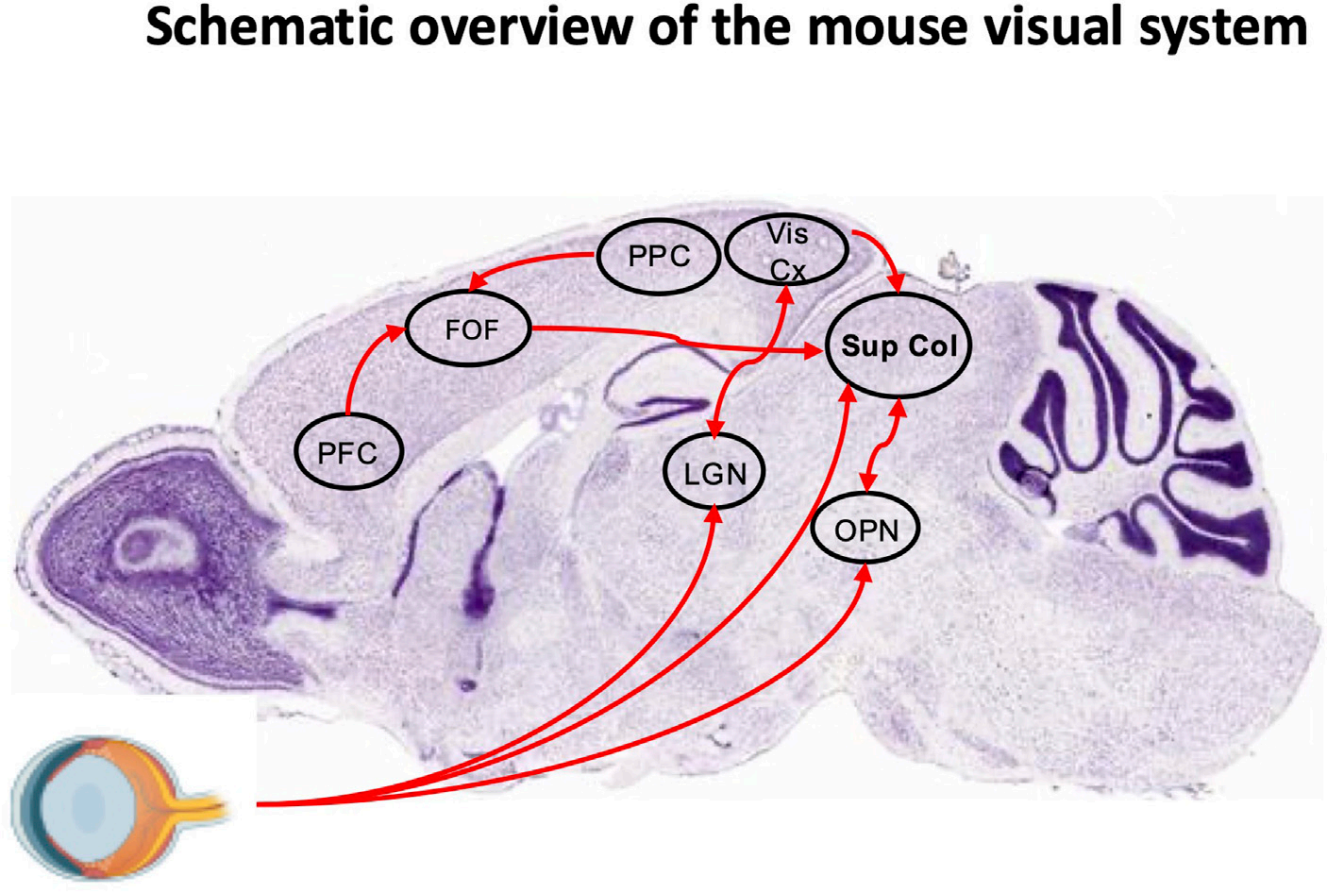
Schematic overview of the mouse visual system. Sagittal section (Nissl staining) from the Allen Brain Atlas overlaid with key regions of the visual system. The frontal orienting field (FOF) receives input from the posterior parietal cortex (PPC) and prefrontal cortex (PFC). Neuronal projections from the FOF, visual cortex (Vis Cx), and olivary pretectal nucleus (OPN), as well as a direct retinal pathway, converge onto the superior colliculus (SC). The lateral geniculate nucleus (LGN) is also indicated.

**FIGURE 4:**
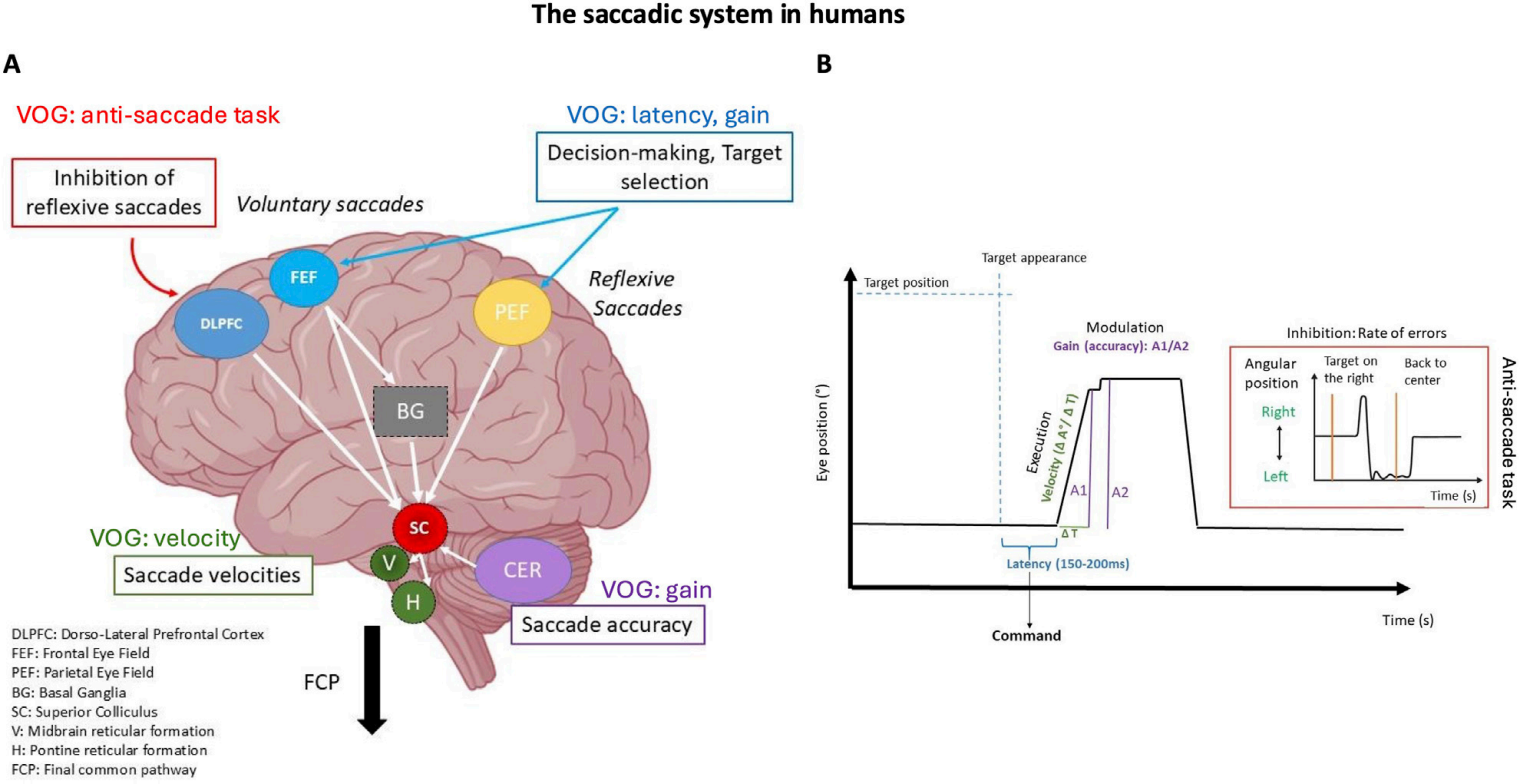
The saccadic system in humans. **(A)** Illustrates the brain regions involved in the command, execution, control, and modulation of saccades. It highlights the specific interaction between cortical and subcortical structures. **(B)** Video Oculography (VOG) trace of a horizontal saccade showing a correspondence between the recorded oculomotor parameters and their relationship with brain structures. The highlighted section shows the anti-saccade trace when there is a failure to inhibit a rightward saccade, resulting in a left-sided error.

In human and rodents several key brain regions orchestrate these eye movements, including (Figures 3, 4):

1. Cortical structures: The fronto-parietal network, consisting of the Frontal Eye Fields (FEF) and parietal eye fields, is integral to visual attention and eye movement control. In humans, the FEF regulates saccades via direct projections to the Superior Colliculus (SC), supports smooth pursuit movements, and coordinates both covert and overt spatial attention (Medendorp et al., 2011; Lane et al., 2012; Vernet et al., 2014; Mirpour and Bisley, 2021). In rodents, the secondary motor cortex, specifically the Frontal Oriented Field (FOF), is considered as a functional homolog of the primate FEF. It projects to the SC, receives input from the posterior parietal cortex and prefrontal areas, and plays a critical role in planning and preparation of orienting movements. Inactivation of the FOF in rodents leads to impaired contralateral orienting, particularly for memory-guided tasks, closely paralleling the role of the FEF in memory-guided saccades in primates. However, while the primate FEF predominantly controls eye movements, the rodent FOF controls combined head and body orienting responses rather than pure ocular saccades, reflecting species differences in oculomotor repertoire (Figure 3). Both systems exhibit delay-period neuronal activity predictive of upcoming movement direction (Bruce and Goldberg, 1985; Erlich et al., 2011; Zahler et al., 2021). The posterior parietal cortex contributes in both species to visuospatial integration and updating spatial representations following eye movements (Duhamel et al., 1992; Medendorp et al., 2011; Klautke et al., 2023). The supplementary eye fields in humans assist in executing eye movements toward memorized locations and sequencing them (Heide et al., 2001; Isoda and Tanji, 2002; Lu et al., 2002) (Figure 4A). A direct rodent supplementary eye fields equivalent is less well defined, though some dorsal medial areas may serve partially analogous functions. The dorsolateral prefrontal cortex (dlPFC) inhibits unwanted reflexive saccades in humans (Pierrot-Deseilligny et al., 2003; 2005; Cameron et al., 2015). Rodents lack a true dlPFC homolog, but their medial prefrontal cortex (mPFC), notably the infralimbic (IL) and prelimbic (PL) cortices, interconnected with the FOF, exerts inhibitory control over orienting and attention. The PL supports goal-directed behavior and memory retrieval, whereas the IL is more engaged in response inhibition, suppression of undesired actions, and habit formation. Both regions are strongly interconnected with the hippocampus, amygdala, striatum, and other cortical regions, enabling flexible learning and adaptive control (Gutman et al., 2017; Capuzzo and Floresco, 2020). Together, PL and IL modulate executive control and response inhibition within broader fronto-parietal circuits. While the FOF directly orchestrates motor planning for orienting actions, the IL and PL provide top-down executive modulation. In primates, the FEF and dlPFC perform broadly analogous roles to the rodent FOF and PL/IL, even though rodents lack a precise dlPFC counterpart. Damage to these interconnected regions disrupts spatial attention and awareness in both species, with rodents showing deficits more in head–body orientation than in gaze control (Zahler et al., 2021). Because of their overlap with higher-order cognitive systems, both human FEF–dlPFC and rodent FOF–PL/IL networks are particularly vulnerable to neurodegenerative processes that impair cognitive control (Ng et al., 2021). This comparative framework underscores how prefrontal regions coordinate attention, memory-guided behavior, and executive control—functions critically impaired in AD. In the frontal cortex, including the FEF, reductions in GABA levels and interneuron dysfunction correlate with impaired cognitive control and attentional regulation (Govindpani et al., 2017; Xu et al., 2020). Similar deficits are seen in the parietal cortex and other neocortical areas (Bai et al., 2015). Specific interneuron subtypes, notably parvalbumin (PV) and somatostatin (SST) cells, appear especially vulnerable: decreased activity and loss of these interneurons have been reported in the medial temporal lobe and medial prefrontal regions, including the rodent PL/IL cortices (Beal et al., 1985; 1986; Saiz-Sanchez et al., 2013; Nava-Mesa et al., 2014; Saiz-Sanchez et al., 2016; Xu et al., 2020). These alterations are tightly linked to network hyperexcitability and cognitive deficits, underscoring the central role of GABAergic dysfunction in AD pathogenesis.
2. Subcortical structures: The basal ganglia form a fundamental subcortical hub, integrating motor, cognitive, and emotional functions through four parallel loops—motor, associative/cognitive, limbic, and oculomotor. Each connects specific cortical regions with basal ganglia substructures and the thalamus, thereby coordinating distinct aspects of behavior (Lanciego et al., 2012). The oculomotor loop is particularly relevant for eye movements and visual attention: frontal and supplementary eye fields project to the caudate nucleus, which relays signals via the substantia nigra pars reticulata (SNr) to the SC. Those neurons provide tonic inhibition of the SC to suppress unwanted saccades; transient pauses in SNr firing disinhibit the SC, enabling controlled gaze shifts (Shires et al., 2010). Thus, the circuit functions as a gatekeeper for oculomotor control. Although substantial GABAergic cell loss is not typically reported in the basal ganglia during AD, synaptic dysfunction at inhibitory terminals may impair regulation of oculomotor pathways (Govindpani et al., 2017). Within the SC, GABAergic signaling suppresses unwanted or reflexive saccades, while transient disinhibition permits planned orienting movements. In this way, the SC integrates diverse oculomotor behaviors—including saccades, smooth pursuit, vergence, and coordinated head movements—and mediates both overt and covert shifts of spatial attention (Lomber et al., 2001; Thier and Ilg, 2005; Krauzlis et al., 2013; Bollimunta et al., 2018; Liu et al., 2022). Essential for gaze control and attentional shifts, the SC appears particularly vulnerable in AD, likely due to its high metabolic demands and extensive connectivity. Although less extensively studied, emerging evidence suggests that impaired inhibitory control within the SC contributes to oculomotor deficits in AD (Dugger et al., 2011; Erskine et al., 2017; Pin et al., 2023). In rodents, the FOF, a functional analog of the primate FEF, projects strongly to the SC and is critical for planning and executing orienting movements involving the eyes, head, and body (Erlich et al., 2011; Zahler et al., 2021). Given the basal ganglia’s central role and broad connectivity, dysfunction within these loops can profoundly disrupt oculomotor control and visual attention, contributing to the deficits observed in neurodegenerative conditions such as AD.
3. Premotor nuclei of the brainstem: These nuclei, including the medial vestibular nucleus, prepositus hypoglossal nucleus, and rostral interstitial nucleus of the medial longitudinal fascicle, are integral to eye movement control and gaze stabilization. They generate and coordinate saccades, smooth pursuit, and vergence movements while integrating sensory input from multiple brain regions. Dysfunction in these areas can contribute to oculomotor abnormalities observed in various neurological disorders (Erlich et al., 2011; Zahler et al., 2021) Despite anatomical differences, these nuclei perform comparable functions across species in integrating motor and sensory signals for gaze stability. Lesions contribute to oculomotor abnormalities across numerous neurological diseases.
4. Autonomic control of pupillary responses: The locus coeruleus (LC) and Edinger-Westphal (EWN) nucleus (EWN) regulate pupillary dilation and constriction, respectively. The LC, through its noradrenergic projections, influences arousal and cognitive function, while the EWN drives the light and near reflexes. Receiving input from the pretectal olivary nucleus, the EWN transmits signals via the oculomotor nerve to the ciliary ganglion and iris sphincter muscle (Mathôt, 2018). Their interaction is complex, with evidence suggesting inhibitory or presynaptic connections between the LC and EWN, possibly involving the SC (Wang and Munoz, 2015). These nuclei integrate signals from multiple brain regions to adjust pupil size based on light, arousal, and cognitive demands. Notably, both are among the earliest affected in neurodegenerative disorders, particularly AD, where pathological changes may emerge before cognitive symptoms (Wang and Munoz, 2015). The LC, a key noradrenergic center, is highly susceptible to oxidative stress and toxic protein accumulation, making it an early target in disease progression (Andrés-Benito et al., 2017; Olivieri et al., 2019; Matchett et al., 2021). Similarly, EWN dysfunction, critical for autonomic regulation, contributes to early pupillary abnormalities in neurodegeneration (Scinto et al., 1999; Mavroudis et al., 2014; Chougule et al., 2019).
5. Cerebellar contributions: The cerebellum fine-tunes eye movements through its connections with the brainstem and other regions. Key areas include the flocculus/paraflocculus for vestibulo-ocular reflex adaptation, nodulus/ventral uvula for otolith-driven eye movements, and dorsal vermis/posterior fastigial nucleus for saccadic accuracy. The cerebellar hemispheres contribute to smooth pursuit and saccades, while the cerebellum also modulates the brainstem neural integrator to maintain stable fixation (Leigh and Zee, 2015). Cerebellar dysfunction is linked to spinocerebellar ataxias, Friedreich’s ataxia, multiple system atrophy (cerebellar type), Parkinson’s disease subtypes, and certain variants of AD (Larner, 1997; Chaudhari et al., 2021; Cheng et al., 2023; Yang et al., 2024). These conditions often lead to motor coordination deficits, balance issues, and abnormal eye movements, underscoring the cerebellum’s essential role in both motor control and cognitive function.

The oculomotor system relies on interconnected neural networks that are vulnerable to neurodegenerative disruptions through both direct and indirect mechanisms. Some neurodegenerative diseases directly impair these networks, leading to observable oculomotor dysfunctions, while others primarily affect cognitive and behavioral circuits, indirectly influencing eye movement control due to the brain’s interconnected nature (Gaymard, 2012; Leigh and Zee, 2015; Shakespeare et al., 2015). This interplay highlights the complex relationship between cognitive, behavioral, and motor functions in the brain (Zola et al., 2013; Dragan et al., 2017; Granholm et al., 2017; Readman et al., 2021). Analyzing eye movement parameters provides valuable diagnostic insights into both the primary impact of neurodegenerative diseases on oculomotor function and the secondary effects of broader neural dysfunctions (Figure 4B).

Overall, eye movement assessments offer a powerful, non-invasive tool for the early detection of neurodegenerative processes, providing valuable insights into disease progression and informing the development of targeted intervention strategies.

### 2.2 Neuronal circuits controlling eye movements and their reliance on precise E/I balance

The visual attention system plays a fundamental role in integrating perception and action, enabling adaptive interactions with the environment. This integration is facilitated by various types of eye movements, which are essential for efficient visual processing and attentional control (Rolfs and Schweitzer, 2022). Among extrinsic eye movements, we distinguish saccades (rapid shifts in gaze between fixation points, fixations) periods of gaze stabilization, smooth pursuit (continuous tracking of moving objects), and vergence movements (adjustments that maintain binocular focus at different distances) (Leigh and Zee, 2015). Intrinsic eye movements, such as the pupillary photomotor reflex and psychosensory response, further enhance visual attention by optimizing retinal image quality, regulating light intake, and reflecting cognitive and emotional states (Mathôt, 2018). These finely tuned mechanisms enable rapid and efficient adaptation to dynamic visual environments, supporting selective attention, spatial awareness, and exploratory behavior.

**FIGURE 5:**
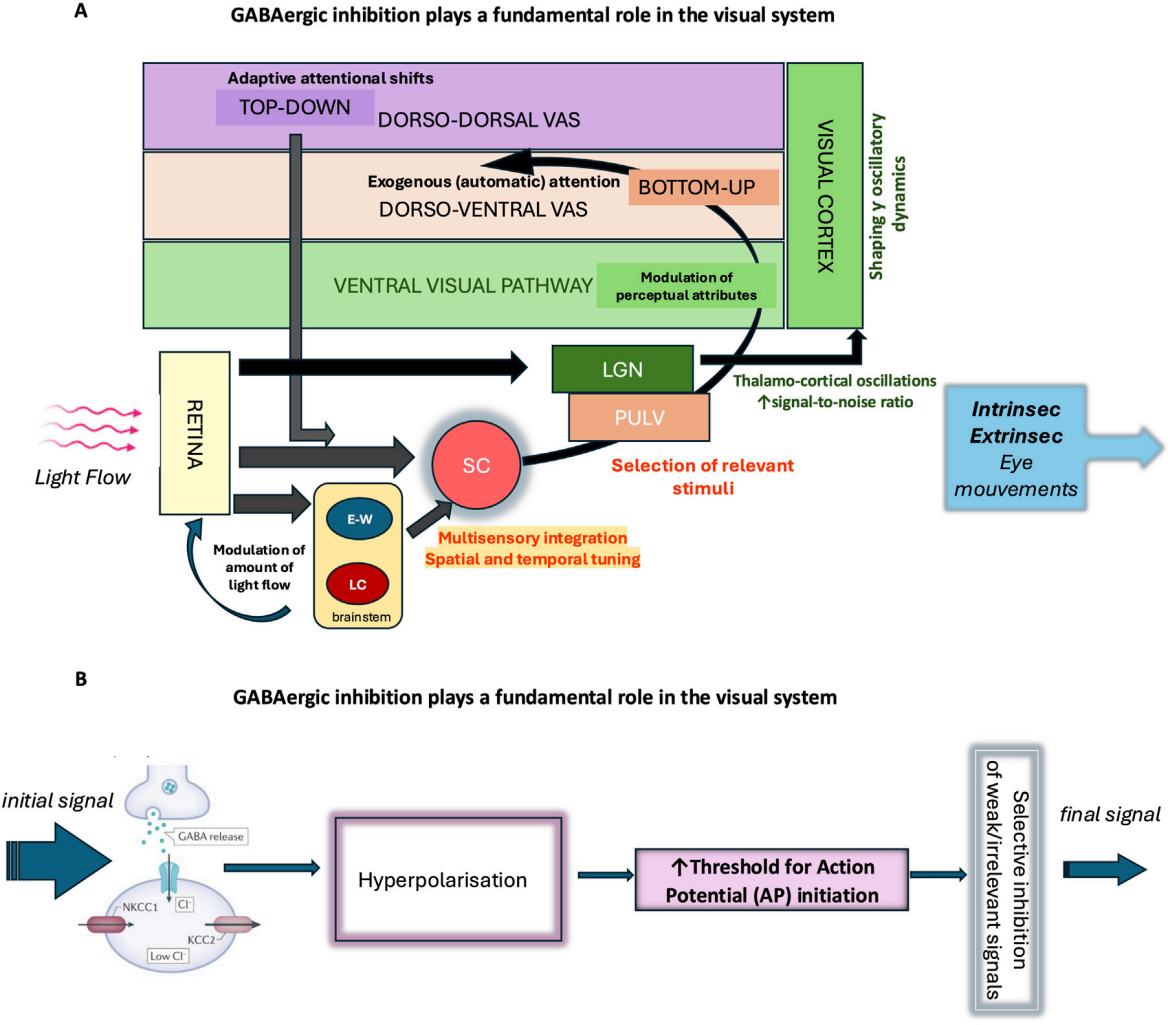
GABAergic inhibition plays a fundamental role in the visual system. **(A)** As the main inhibitory neurotransmitter in the central nervous system, GABA enables the fine regulation of neuronal excitability and actively participates in the selection of visual information. Its action begins early, at the level of the retina, where it modulates neuronal circuits involved in the initial processing of light signals and contributes to the spatiotemporal encoding of visual information. The regulation of pupil size mainly depends on autonomic circuits within the brainstem such as the Edinger-Westphal nucleus (EWN) and Locus coeruleus (LC). Throughout the stages of visual information integration, GABAergic inhibition shapes and modulates signal transmission, enhancing contrast and the selectivity of neuronal responses. In the ventral visual pathway, it contributes to object formation and recognition (percept formatting), while in the dorsal pathway, it is involved in the selection of salient information and the management of automatic visual attention, in interaction with dorso-ventral attentional control networks. GABAergic inhibition also plays a role in the voluntary orientation of visual attention (dorso-dorsal visual attentional system) according to behavioral and homeostatic needs and contributes to the overall regulation of visual behavior. The analysis of oculomotor responses—whether intrinsic (pupillary responses) or extrinsic (eye movements) —provides an objective tool for studying these mechanisms of integration and regulation of visual information. SC (Superior Colliculus), LGN (lateral geniculate nucleus), PULV (pulvinar), VAS (visual attention system). **(B)** GABAergic interneurons receive excitatory input and release GABA, which activates GABA_A_ receptors on target neurons. Activation of these receptors causes an influx of Cl^−^ ions, hyperpolarizing the membrane and making it more difficult to generate an action potential. This mechanism acts as a filter: it selects strong or relevant signals and prevents the propagation of weak or irrelevant signals, thereby ensuring precise control of neuronal transmission. This GABAergic filtering thus enables the active selection of signals transmitted within neural circuits, preventing overload and promoting the accuracy of brain responses.

The interaction between visual attention and eye movements is bidirectional: attention directs eye movements toward areas of interest, while eye movements influence attentional allocation (Sheliga et al., 1994; Kowler et al., 1995; Deubel and Schneider, 1996; Hutton, 2008; Ibbotson and Krekelberg, 2011; Souto and Kerzel, 2021). This dynamic relationship enables efficient visual exploration and rapid adaptation to environmental changes. At its core, this system maintains a balance between two complementary processes (Katsuki and Constantinidis, 2014) (Figure 5A): i). Bottom-up (stimulus-driven) processes, triggered by the intrinsic properties of visual stimuli; ii). Top-down (goal-directed) processes, guided by higher cognitive and motivational factors. This balance is essential for two key reasons. First, it sustains visual attention on ongoing tasks, ensuring continuity and efficiency of action. Second, it enables rapid attentional shifts in response to environmental changes, optimizing adaptability. The system’s efficiency relies on a finely tuned interplay between inhibitory and excitatory neural circuits. Various neuronal populations, each with distinct firing patterns and transmission modes, contribute to this regulation. In visual attention systems, GABAergic inhibition is especially critical for two fundamental mechanisms (Figure 5B):

#### 2.2.1 Selection of relevant information

GABAergic transmission plays a crucial role in sensory processing by efficiently gating and integrating information, suppressing background noise, and refining visual perception.

• Retinal Circuitry: In the retina, GABAergic transmission modulates neural activity at multiple levels, serving as a crucial mechanism for sensory gating and the selection of relevant visual information. Among GABA receptor subtypes, GABA_C_​Rs (ρ-subunit–containing) are concentrated on bipolar cell terminals, where they generate sustained tonic inhibition characterized by slow kinetics and high GABA sensitivity. Functionally, GABA_C_​-mediated inhibition contributes to gain control and signal-to-noise optimization by suppressing background activity and preventing saturation of bipolar responses, thereby enhancing contrast sensitivity and visual acuity (Wang et al., 2007; Popova, 2014; Matsumoto et al., 2025; Medina Arellano et al., 2025). By contrast, GABA_A_​Rs, widely expressed on retinal ganglion cells (RGCs) and other retinal neurons, mediate fast, phasic inhibition that dynamically shapes RGC excitability and firing patterns. This rapid inhibition supports precise temporal tuning, direction selectivity, and motion detection in the inner retina (Wang et al., 2007; Okumichi et al., 2008). Together, the complementary actions of GABA_C_​ and GABA_A_​Rs allow the retina to filter, refine, and transmit selected visual information efficiently, ensuring accurate visual perception.
• Superior Colliculus: In the superior colliculus, GABAergic inhibition is vital for shaping appropriate behavioral and physiological responses. In sensory processing, it refines topographically aligned visual, auditory, and somatosensory inputs, facilitating precise multisensory integration (Behan et al., 2002). In motor command generation, GABAergic circuits regulate orienting behaviors such as saccadic eye movements, with tonic inhibition from the SNr playing a crucial role (Kaneda et al., 2008). Moreover, GABAergic inhibition fine-tunes the spatial and temporal properties of collicular responses, ensuring appropriate E/I balance and preventing excessive activation (Behan et al., 2002; Kaneda et al., 2008).
• Thalamic Visual Pathways and Their Role in Visual Attention: Visual information reaches the cortex through two main thalamic pathways that are regulated by GABAergic inhibition, each playing distinct roles in visual attention:
o Lateral Geniculate Nucleus Pathway

The lateral geniculate nucleus is the primary sensory relay nucleus in the thalamus, transmitting retinal signals to the primary visual cortex (V1). GABAergic inhibition dynamically filters and sharpens visual signals, supporting selective and sustained visual attention by maintaining thalamo-cortical oscillations and optimizing signal-to-noise ratio (Kim et al., 1997; Ye et al., 2017; Klein et al., 2018).

o Colliculo-Pulvinar Pathway

An alternative visual route involves the SC sending processed visual information to the pulvinar nucleus, which then projects to visual and parietal cortical areas involved in spatial attention and gaze control. GABAergic inhibition within the SC finely regulates the flow of salient visual information to the pulvinar, enabling rapid detection of visual targets and flexible reorientation of spatial attention. This pathway is critical for exogenous (automatic) attention and the selection of relevant stimuli in complex environments (Berman and Wurtz, 2011; Soares et al., 2017; Fang et al., 2020).

Together, these pathways, through distinct GABAergic inhibitory mechanisms, enable the brain to balance focused, selective attention with rapid, flexible orienting responses, ensuring efficient processing of visual information according to behavioral demands.

• Gamma Oscillations in the primary visual cortex (V1): The density of GABA_A_Rs in the human V1 correlates positively with gamma peak frequency and negatively with gamma amplitude, highlighting their role in shaping gamma oscillatory dynamics essential for efficient visual processing (Kujala et al., 2015).
• Cortical Networks and Visual Perception: Recent findings reveal that GABA’s influence on visual perception is region-specific. In the parietal cortex, GABA levels correlate with size perception, while in the occipital cortex, they influence orientation perception. This suggests that GABA functions as a precise modulator of distinct perceptual attributes rather than merely exerting global inhibition, underscoring its sophisticated role in shaping visual experience through specialized cortical networks (Song et al., 2017).

#### 2.2.2 Attentional flexibility

GABAergic inhibition plays a critical role in adaptive behavior by facilitating the interruption of ongoing actions when necessary, enabling the reallocation of attentional resources to more contextually appropriate tasks.

• Neural Dynamics and Working Memory: In higher-order cortical regions, GABAergic inhibition shapes neural dynamics and supports working memory performance. The density of GABA_A_Rs in these areas predicts reaction times in working memory tasks and correlates positively with gamma oscillation peak frequency while negatively with BOLD amplitude. These interactions contribute to dynamic complexity and spatiotemporal flexibility in cortical networks, crucial for adaptive attentional shifts (Kujala et al., 2024).

Overall, the visual attention system, in coordination with eye movements, optimally synchronizes perception and action through complex regulatory mechanisms. This sophisticated interplay serves a crucial adaptive function, enabling organisms to meet homeostatic needs, adapt to dynamic environmental demands, process relevant visual information while filtering out distractions, and rapidly shift focus to salient stimuli. The GABAergic system contributes significantly to this balancing process by modulating neural activity across multiple levels of visual processing. Through its inhibitory action, GABA refines neuronal response properties from the retina to higher cortical areas, enhancing signal selectivity, optimizing sensory integration, and ensuring efficient attentional control.

### 2.3 Alterations in oculomotor circuits reflect broader impairments in brain function

Neurodegenerative diseases selectively target interconnected functional networks in the brain, progressively disrupting their structure and function (Seeley et al., 2009; Drzezga, 2018). Oculomotor networks exemplify this vulnerability, illustrating how complex neural circuits are systematically affected by neurodegeneration (Gorges et al., 2018). The susceptibility of these networks stems from their tight functional integration, where dysfunction in one region can cascade through multiple functional domains. Maintaining precise synaptic regulation within these networks is critical for preventing neuronal overstimulation and ensuring the production and recycling of growth factors essential for neuronal survival (Palop et al., 2007; Palop and Mucke, 2010). A delicate balance between regulatory and stress factors maintains network stability. Regulatory mechanisms mitigate the harmful effects of stressors, preserving neural circuit integrity. However, when this balance is disrupted, aberrant neuronal discharge patterns emerge, promoting maladaptive network dynamics that drive the spread of pathological behavioral changes (Kazim et al., 2021; Karimani et al., 2024). This propagation is driven by an imbalance between excitatory and inhibitory activity, triggering maladaptive network dynamics that amplify dysfunction and accelerate disease progression.

Traumatic brain injury (TBI) has emerged as a critical model for understanding how acquired brain vulnerabilities disrupt neural homeostasis, particularly through E/I imbalance. Moderate-to-severe or repeated mild TBI elevates dementia risk up to 4.5-fold and accelerates cognitive decline by years (Graham and Sharp, 2019; Mielke et al., 2022), with mechanistic overlaps to AD including shared chloride homeostasis disruption and inhibitory neurotransmission deficits. One proposed link is the persistent dysregulation of chloride transporters, leading to depolarizing GABAergic signaling and prolonged network hyperexcitability (Hui et al., 2016; Zhang et al., 2017; Tessier et al., 2023; Caccialupi Da Prato et al., 2025; Hochstetler et al., 2025). A study investigating vascular pathology post-TBI identified early Aβ aggregation and reduced NOTCH3 expression in vascular smooth muscle cells, implicating cerebrovascular dysfunction as a key driver of AD-related changes (Özen et al., 2025). Given that chloride dysregulation contributes to blood-brain barrier impairment, these findings suggest a pathological trajectory linking TBI-induced neurovascular damage to AD-related neurodegeneration. Further supporting this connection, studies in TBI models demonstrate that NKCC1 inhibition alleviates blood-brain barrier disruption and reduces neuroinflammation by suppressing the NF-κB/NLRP3 signaling pathway (Bergauer et al., 2022; Zhang et al., 2025). Additionally, individuals with a history of repeated mild TBI exhibit increased markers of intracellular chloride homeostasis dysregulation and elevated NKCC1 expression, leading to persistent excitotoxicity and cognitive decline (Li J. et al., 2024) (Figure 5). Persistent chloride imbalances in TBI models have also been linked to long-term synaptic dysfunction and neuronal circuit remodeling, mirroring changes observed in early stage of AD (Srinivasan and Brafman, 2021; Capsoni et al., 2022; Lam et al., 2022; Barbour et al., 2024; Panayi et al., 2024; Zhang et al., 2024; Sun et al., 2025). A growing body of evidence suggests a shared pathophysiological mechanism between TBI-induced E/I imbalance and the eye movement abnormalities seen in AD. TBI leads to persistent disruptions in chloride homeostasis and GABAergic transmission, which may underlie the oculomotor deficits observed in both TBI and AD. Similar to AD, the downregulation of KCC2 and upregulation of NKCC1 following TBI result in intracellular chloride accumulation, weakening GABAergic inhibitory transmission. In both conditions, impaired inhibitory control within the SC and prefrontal cortex results in delayed saccades, deficits in memory-guided eye movements, and reduced visual tracking accuracy (Tyler et al., 2015; McDonald et al., 2022; Bell et al., 2023; Cade and Turnbull, 2024). Additionally, disruptions in gamma oscillations, closely linked to GABAergic dysfunction, further compromise oculomotor coordination. These findings indicate that TBI may accelerate neurodegeneration by destabilizing inhibitory networks crucial for eye movement control. Future studies should investigate whether normalizing chloride homeostasis in TBI survivors could mitigate Alzheimer’s-related neurodegeneration, potentially unveiling dual-purpose therapeutic strategies. This approach gains urgency from recent findings showing that TBI-induced chloride dysregulation persists for years post-injury, creating a latent window for intervention.

A mechanistic understanding of how E/I imbalance propagates network instability is critical for developing treatments that preserve and promote neural integrity across disease stages. Importantly, the observed interplay between oculomotor deficits and cognitive decline in both TBI and AD underscores the necessity of systems-level interventions, therapies addressing not just molecular targets but distributed neural network functionality.

### 2.4 Eye movement deficits in AD: neural hyperexcitability and network desynchronization from E/I imbalance

**FIGURE 6:**
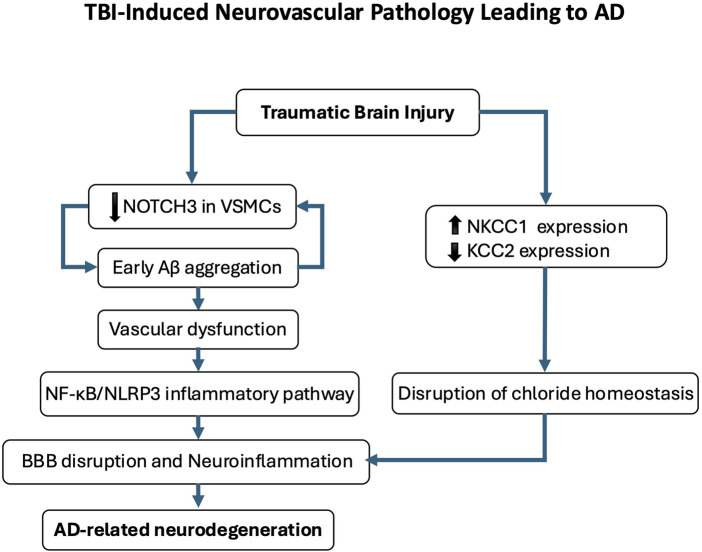
Traumatic brain injury (TBI)-induced neurovascular pathology leading to Alzheimer’s disease (AD). This schematic illustrates the proposed pathological cascade linking TBI to AD-related neurodegeneration. Following TBI, early cerebrovascular alterations include amyloid-β (Aβ) deposition and reduced NOTCH3 expression in vascular smooth muscle cells, contributing to vascular dysfunction. Disruption of chloride homeostasis, characterized by NKCC1 upregulation and KCC2 downregulation, compromises blood-brain barrier (BBB) integrity and promotes neuroinflammation. This neurovascular dysfunction activates the NF-κB/NLRP3 inflammatory pathway, further exacerbating neuronal injury and cognitive decline. Repeated mild TBIs reinforce this trajectory by sustaining chloride dysregulation and perpetuating a pro-degenerative microenvironment, ultimately contributing to AD-like pathology.

Gamma oscillations are essential for neural communication and cognitive function, serving as a dynamic gating mechanism that enhances information routing, memory processing, attention, and overall cognitive performance (Guan et al., 2022). These high-frequency brain waves are highly sensitive to neuronal network dysfunction, making them valuable early indicators of potential neurodegenerative processes, often preceding clinical symptom onset (Mably and Colgin, 2018). The generation and maintenance of gamma oscillations rely on a finely tuned balance between E/I inputs, enabling coherent oscillatory patterns while preserving flexibility to adapt to environmental demands (Orekhova et al., 2017) (Figure 6A). However, in AD, this balance is progressively disrupted, leading to heightened neuronal excitability and cortical hyperexcitability (Maestú et al., 2021). Multiple factors contribute to this imbalance, including Aβ and tau accumulation, dysfunction of inhibitory GABAergic interneurons, altered glial cell activity, and genetic predispositions such as the ApoE4 genotype (Kurosinski and Götz, 2002; Wishart et al., 2006; Cheng et al., 2020; Mattson, 2020; Umpierre and Wu, 2021; Sun et al., 2023). The E/I imbalance in AD is characterized by a preferential impairment of local inhibitory connections, primarily mediated by GABAergic interneurons, relative to excitatory ones. Consequently, individuals with MCI and AD exhibit weakened neural connections, leading to a progressive decoupling of neural populations.

**FIGURE 7:**
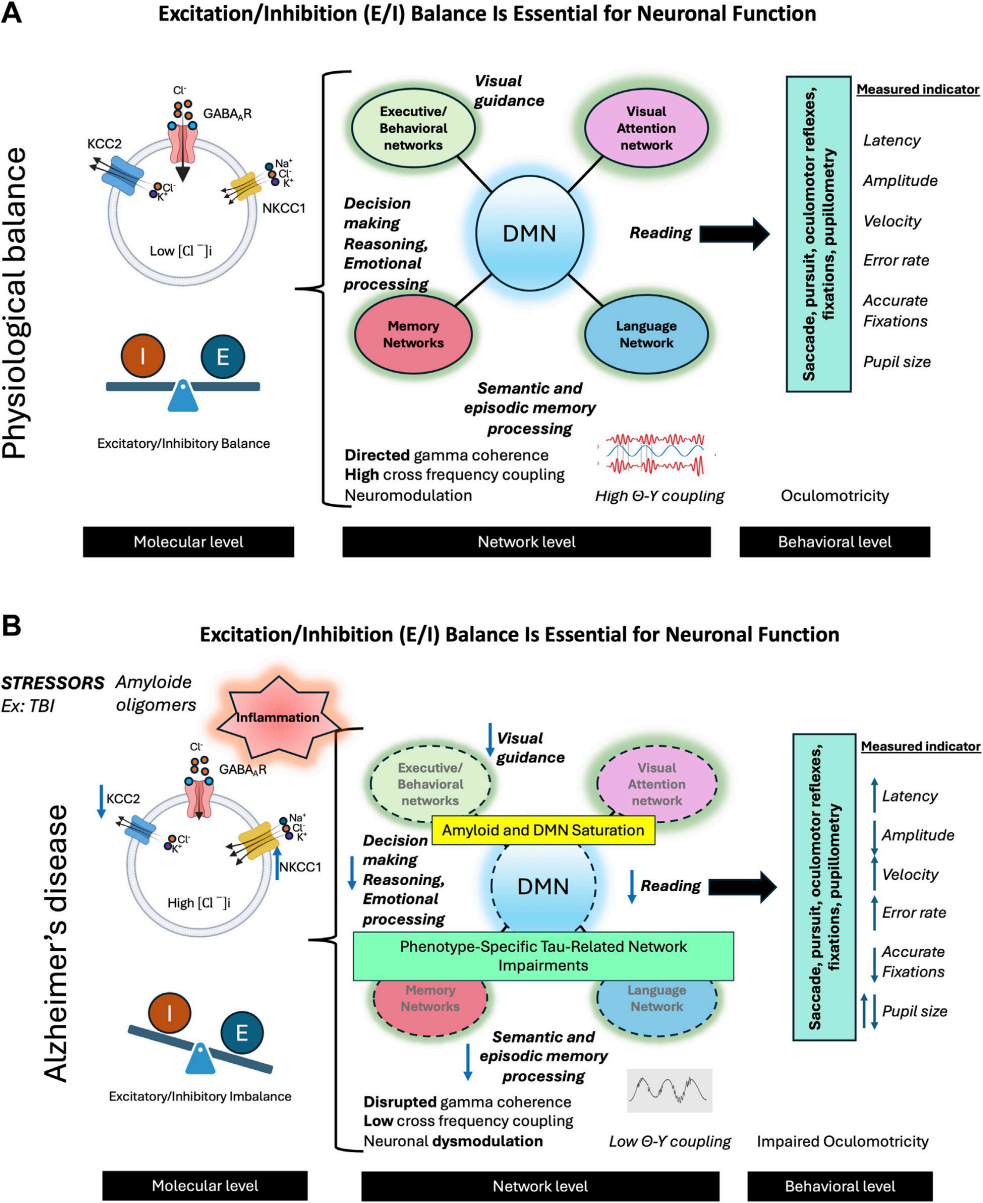
The balance between excitation and inhibition (E/I) in the brain is crucial for optimal neuronal functioning. **(A)** In the physiological state, this balance is maintained by several mechanisms, primarily through the action of GABAergic neurons and the activity of the KCC2 cotransporter. The latter plays an essential role in extruding chloride ions (Cl^−^) from neurons, thus counterbalancing the action of NKCC1, which brings these ions into the cells. This effective inhibition allows neural networks to enter into oscillatory activity, promoting network activity in the gamma band and the synchronization of theta-gamma activity. These oscillations are crucial for neuromodulation and optimal activity of networks supporting complex cognitive functions such as visual attention, language, memory, and behavior. The E/I balance also facilitates effective interaction between the default mode network (DMN) and networks specifically involved in various cognitive functions. The activity of these networks can be evaluated by studying oculomotor behavior during different cognitive tasks. The combined analysis of oculomotor parameters such as latencies, velocities, amplitudes, and error rates provides an interesting insight into the functioning of neural networks. **(B)** In Alzheimer’s disease (AD), various stress factors, including traumatic brain injuries (TBI), trigger a cascade of dysfunctions. We observe neuroinflammation leading to a decrease in KCC2 activity, the formation of beta-amyloid oligomers, an imbalance in the E/I balance, an alteration of gamma activity, and a desynchronization of theta-gamma activity, leading to neural network dysfunction. These changes result in amyloid saturation of the DMN, common to different AD phenotypes, as well as the propagation of lesions specifically associated with clinical phenotypes (visual, language, memory, behavioral) linked to Tau pathology. These early alterations can be detected by studying oculomotor behavior during various cognitive and oculomotor tasks. We can thus observe the alteration of different oculomotor parameters, offering a window into the underlying neuronal changes.

The disruption of GABAergic inhibition is particularly critical, as GABA is essential for stabilizing gamma oscillations (Limon et al., 2012). Studies have highlighted the role of diminished GABAergic function in AD pathogenesis and its consequences on brain oscillations, particularly in resting-state networks where an elevated functional E/I ratio correlates with cognitive decline (Palop et al., 2007; Bi et al., 2020; Xu et al., 2020; Jiménez-Balado and Eich, 2021; Lauterborn et al., 2021; Scaduto et al., 2023). Aβ and tau accumulation further exacerbate this imbalance, promoting neuronal hyperactivity and impairing synaptic plasticity, ultimately disrupting gamma oscillations (Chapman et al., 1999; Minkeviciene et al., 2009; Targa Dias Anastacio et al., 2022). As gamma activity declines, neural circuit communication deteriorates, contributing to both amnestic and non-amnestic AD symptoms (Murty et al., 2021; Traikapi and Konstantinou, 2021) (Figure 7; Box 1).

Box 1 **Key Points on Oculomotor Disturbances in Alzheimer's Disease Phenotypes.**

**Typical Phenotype (Memory-Onset AD)**

Neural Network Disruption and Clinical Features:

Neurodegeneration in typical memory onset Alzheimer's disease originates in the ventral anterior temporal network, disrupting non-contextual memory encoding before spreading to dorsal medial temporal regions and impairing contextual/spatial processing. This progression correlates with connectivity loss within the default mode network (DMN) (Kahn et al., 2008; Zhou et al., 2008; Didic et al., 2011; Gour et al., 2011; Koronyo et al., 2013; Jones et al., 2016).

Oculomotor Measures:

In AD, eye-tracking metrics - including reduced stable fixation duration, increased saccadic latency, and impaired visual pursuit (Garbutt et al., 2008; Molitor et al., 2015)-combined with pupillometry may offer objective measures of neural network dysfunction. These techniques map attention allocation through the dorsal (spatial processing) and ventral (object recognition) visual pathways during memory-related tasks (Corbetta, 1998). These tools quantify subtle alterations in gaze patterns and pupil responses, offering a window into preclinical or early-stage network dysfunction.

**Visual Variant (Posterior Cortical Atrophy or PCA)**

Neural Network Disruption and Clinical Features:

PCA is a posteriorly shifted neurodegenerative syndrome with predominant parietal/occipital atrophy affecting ventral (object recognition) and dorsal (spatial processing) visual networks, most commonly linked to AD (Crutch et al., 2017; Chapleau et al., 2024).

Oculomotor Measures:

Eye movement patterns reflect posterior neuro-visuo-cortical vulnerability, critical areas for visuospatial attention: Impaired visual pursuit (reduced accuracy in tracking moving objects), Saccadic deficits (increased latency and errors in voluntary gaze shifts), fixation instability (difficulty maintaining steady gaze) (Shakespeare et al., 2015; Pin et al., 2023).

**Language Variant (Logopenic variant of primary progressive aphasia or lvPPA)**

Neural Network Disruption and Clinical Features

The IvPPA in AD is characterized by neurodegeneration within the dorsal phonological network, which encompasses the left posterior superior and middle temporal gyri and the inferior parietal lobule (Gorno-Tempini et al., 2004, 2011). This degeneration disrupts phonological encoding, retrieval, and working memory, manifesting as impaired sentence repetition, phonemic paraphasias, and lexical retrieval deficits.

Oculomotor Measures:

Oculomotor profiling in IVPPA leverages eye-tracking during language tasks to quantify reading dysfluency (prolonged fixations on phonologically complex words) and impaired comprehension monitoring (reduced anticipatory saccades). Explicitly links metrics to phonological processing and working memory, core deficits in 1vPPA (Readman et al., 2021; Nelson et al., 2023).

**Frontal Variant**

Neural Network Disruption and Clinical Features

The frontal variant of Alzheimer's disease (fvAD) arises from early dysfunction in fronto-subcortical circuits, driving executive and behavioral deficits (e.g., apathy, impulsivity) that precede memory decline (Larner, 1997; Bruno et al., 2023).

Oculomotor Measures:

Eye-tracking and pupillometry - including antisaccade errors (to a lesser extent), prolonged saccadic latency, and distinct task-evoked pupillary responses - provide a non-invasive method to differentiate frontal variant Alzheimer's disease (fvAD) from behavioral variant frontotemporal dementia (bvFTD) (Lage et al., 2021). These metrics quantify disease-specific attentional and emotional processing deficits, which correlate with divergent fronto- subcortical network impairments: fvAD disrupts fronto-subcortical circuits and the default mode network (DMN), whereas bvFTD primarily affects the salience network (SN) and subcortical modules (Ng et al., 2021).

Cross-Cutting Insights

Oculomotor disturbances mirror Alzheimer's neurobiological heterogeneity. They provide non-invasive early biomarkers, complementary to neuroimaging. Critical for differential diagnosis between Alzheimer's phenotypes and other dementias

Interestingly, these neurophysiological alterations manifest in oculomotor abnormalities. The oculomotor system, which relies on precise neural coordination, serves as a valuable indicator of broader neural dysfunction in AD. Eye movement impairments correlate with cognitive decline and disease progression, with measurable changes in oculomotor parameters reflecting underlying network disruptions linked to E/I imbalance and impaired gamma oscillations (Riek et al., 2023; Tokushige et al., 2023; Qi et al., 2024) (Figure 6B). This relationship underscores the potential of oculomotor metrics as biomarkers for AD, bridging cellular-level dysfunction with observable clinical manifestations and offering insight into the complex interplay between neurophysiological alterations and behavioral outcomes (Figure 7).

### 2.5 Synaptic plasticity alterations in AD and their impact on memory-guided saccades

Alzheimer disease is marked by profound synaptic plasticity alterations, closely linked to oculomotor dysfunction, particularly impairments in memory-guided saccades. Synapse loss is an early and defining feature of AD, strongly correlating with dementia severity and preceding both neuronal loss and cognitive decline (Shankar and Walsh, 2009). This dysfunction systematically disrupts brain regions essential for oculomotor control, impairing the neural circuits responsible for precise eye movements.

Memory-guided saccades directed toward remembered spatial targets depend on coordinated activity across a distributed neural network. Key regions include the posterior parietal cortex (spatial processing), dorsolateral frontal cortex (motor planning), prefrontal cortex (working memory), hippocampus (memory consolidation), and subcortical structures such as the SC (Pierrot-Deseilligny et al., 1995; Sugiura et al., 2004; Vericel et al., 2024). These areas integrate visual information, retain spatial memory, and execute precise eye movements through dynamic synaptic interactions.

In AD, progressive neurodegeneration disrupts this network, causing oculomotor deficits such as inaccurate saccades and impaired spatial memory. These deficits mirror broader synaptic dysfunction, particularly in synaptic plasticity-a process critical for learning and memory. Notably, GABA_A_Rs containing the α5 subunit emerge as pivotal regulators of plasticity. By fine-tuning excitatory neuron activity, these receptors modulate the balance between long-term potentiation and long-term depression, mechanisms underlying adaptive neural rewiring (Engin et al., 2013; Zhu et al., 2023). Dysfunctional GABAergic signaling in AD reduces cognitive flexibility and destabilizes memory retention, exacerbating both motor and cognitive decline that can be investigated through oculomotor biomarkers, such as aberrant memory-guided saccades. This interplay highlights how synaptic plasticity deficits, driven by impaired GABA_A_Rs activity, contribute to the pathophysiology of AD (Wolff et al., 1993; Schmidt-Wilcke et al., 2018). Importantly, these GABAergic mechanisms are closely influenced by the function of KCC2. Beyond its canonical role in maintaining low intracellular chloride concentrations necessary for effective inhibitory signaling, KCC2 has emerged as a critical regulator of neuronal structure and function.

The co-transporter KCC2 engages in chloride-independent signaling pathways that modulate cytoskeletal dynamics and synaptic connectivity. Notably, KCC2 interacts with the Rac1/Cdc42 guanine nucleotide exchange factor β-PIX, thereby inhibiting its activity and attenuating downstream Rac1 signaling. This suppression limits the phosphorylation of cofilin-1, an actin-depolymerizing factor, and promotes dynamic actin remodeling within dendritic spines, a process essential for spine morphogenesis and the stabilization of glutamatergic synapses (Chamma et al., 2012; Llano et al., 2015). Loss of KCC2 disrupts this regulatory pathway, resulting in elevated levels of phosphorylated (inactive) cofilin-1, excessive stabilization of actin filaments, and reduced spine motility. These alterations compromise excitatory synaptogenesis and underscore the structural function of KCC2 as a modulator of synaptic architecture (Llano et al., 2015; Côme et al., 2019). Importantly, studies employing conditional deletion of KCC2 in adult glutamatergic neurons have demonstrated deficits in both spatial and nonspatial memory, suggesting that KCC2 contributes to cognitive processes via both inhibitory regulation and maintenance of excitatory synaptic integrity. Interestingly, while long-term potentiation at the dendritic level remains preserved, enhanced excitatory postsynaptic potential-to-spike coupling indicates a shift in E/I balance, likely due to impaired GABAergic inhibition resulting from disrupted chloride homeostasis (Kreis et al., 2023). Together, these findings position KCC2 as a multifunctional protein at the interface of ion transport, cytoskeletal remodeling, and synaptic function. Its dual role, mediating inhibitory efficacy through chloride extrusion and supporting excitatory connectivity through actin-dependent mechanisms, places KCC2 at the center of mechanisms governing synaptic plasticity and memory formation.

This dynamic balance between long-term potentiation and long-term depression enables flexible and efficient information processing, supporting higher-order cognitive functions and adaptive behavior. In AD, disruption of this balance contributes to impaired memory-guided saccades, further underscoring the role of synaptic plasticity deficits in disease progression.

### 2.6 Oculomotor biomarkers that may be affected by abnormalities in GABAergic signaling in AD

GABAergic transmission plays a pivotal role in the generation and control of saccades, influencing velocity, latency, and targeting precision through inhibitory mechanisms in the SC. Increased GABAergic inhibition in this structure has been associated with reduced saccade velocity, prolonged latency, and decreased accuracy in target acquisition. Additionally, GABA contributes to fixation stability, with GABAergic neurons in the rostral SC playing a crucial role in maintaining steady fixation (Villalobos et al., 2018). The competition between automated and voluntary saccade commands is also modulated by GABAergic neurons across various brain regions, particularly in the basal ganglia, where inhibitory control mechanisms help regulate saccadic initiation and suppression (Coe and Munoz, 2017). Abnormal GABA transmission can result in difficulty suppressing saccades to peripheral targets during fixation, particularly in tasks that require voluntary control over reflexive eye movements, such as the anti-saccade task. In this paradigm, which demands the suppression of automatic saccades toward a sudden stimulus, GABAergic inhibition is critical for preventing express latency direction errors (Coe and Munoz, 2017). The gap effect, a well-documented phenomenon in pro-saccade latencies, further suggests a common GABAergic mechanism in saccade programming, emphasizing the role of inhibitory control in the timing of reflexive eye movements (Mize, 1992; Behan et al., 2002; Carrasco et al., 2011; Essig et al., 2021). Pharmacological modulation of GABAergic activity also affects saccade dynamics. Benzodiazepines, for example, can reduce peak saccade velocity while simultaneously improving the precision of anticipated eye position beliefs (Bittencourt et al., 1981). Beyond saccades, GABA influences smooth pursuit eye movements, affecting both initiation and maintenance. Inhibitory signaling within the brainstem plays a role in pursuit onset timing and may also impact the ability to suppress intrusive saccades during smooth tracking (Blazquez and Yakusheva, 2015).

**FIGURE 8:**
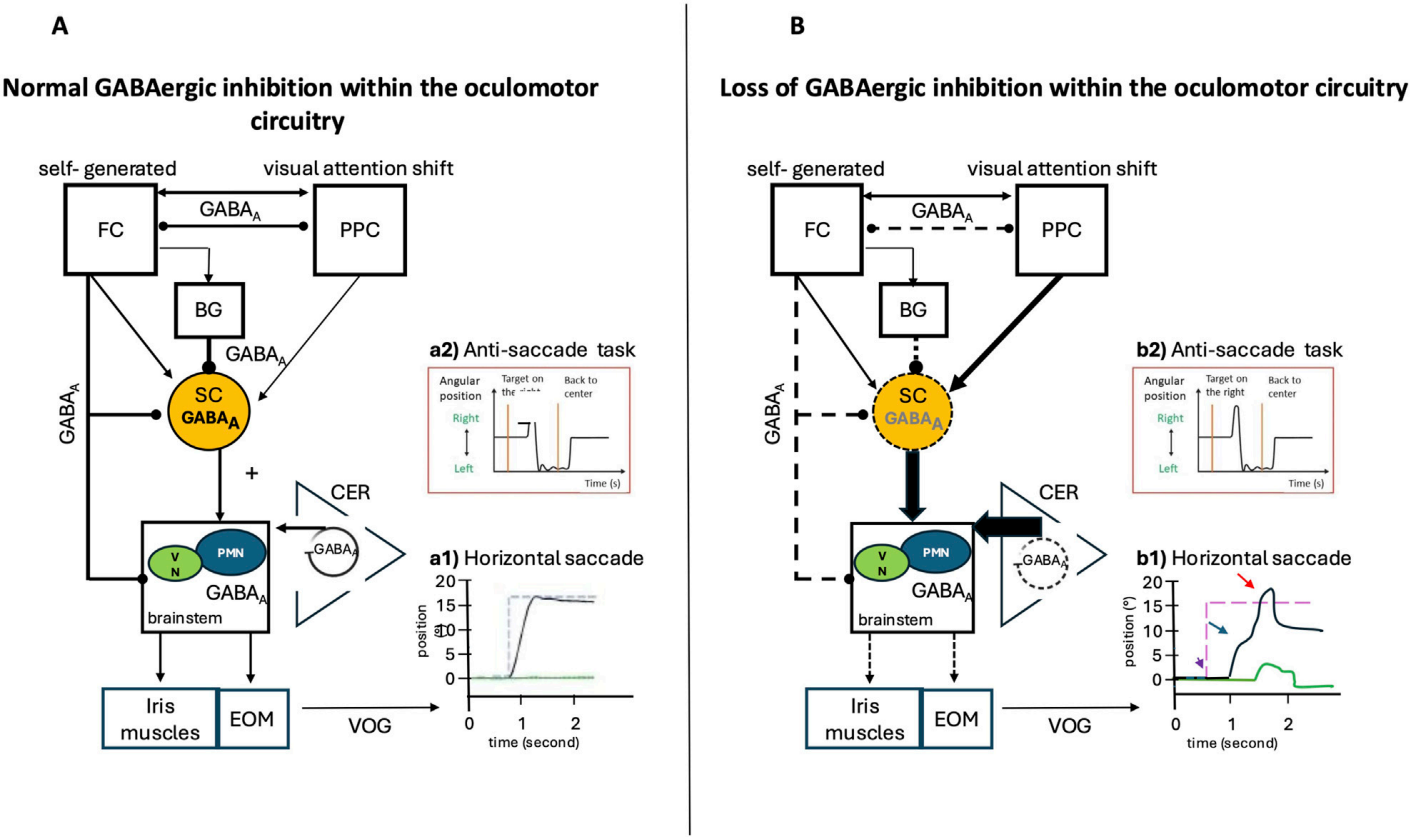
Normal GABAergic Inhibition within the Saccade Network and Possible Impairments in Case of Loss of Inhibition: Video Oculography (VOG) Findings. Eye position is plotted on the x-axis, and the y-axis depicts the corresponding time in seconds. Black lines indicate the horizontal eye position; green traces illustrate the vertical eye position, and blue dashed lines show the position of the target (desired eye position). **(A)** Inputs from the posterior parietal cortex (PPC) and frontal cortex (FC), via direct or indirect pathways through the basal ganglia, are sent to the superior colliculus (SC). The SC is a pivotal structure, integrating and relaying commands from the cerebral cortex to the premotor brainstem saccadic nuclei, then to the extrinsic oculomotor muscles (EOM), and influencing the vegetative pupil motor nuclei (VN) and the iris muscles, which are responsible for intrinsic oculomotor function. The cerebellum (CER) exerts a modulatory function and controls the precision of saccades. GABAergic inhibition plays a key role in the control of rapid eye movements (saccades) by modulating the excitability of the involved neural circuits, particularly in the cerebellum, the superior colliculus, and the basal ganglia. Reciprocal GABAergic inhibition between frontal and posterior parietal structures can influence saccade latency by modulating the balance between excitation and inhibition within oculomotor circuits. **(B)** Loss of GABAergic inhibition alters neural circuits by increasing excitatory output within various cortical and subcortical structures. (*A1*) VOG record of a normal horizontal saccade trajectory and normal inhibitory control in the (*A2*) anti-saccade task. (*B1*) VOG records in the case of loss of GABAergic inhibition show several impairments: Amplitude and gain: Reduced GABAergic inhibition increases the response of cerebellar neurons during saccades, altering their amplitude and directional selectivity (red arrow), Velocity and duration: GABAergic dysfunction in the superior colliculus can decrease peak saccade velocity and prolong their duration (blue arrow), Latency and initiation: The release of tonic GABAergic inhibition (from the basal ganglia to the superior colliculus) is a key trigger for saccade initiation. Excess inhibition delays or prevents saccades, while disinhibition facilitates rapid initiation (purple arrow). (*B2*) Loss of inhibition in frontal regions leads to errors in anti-saccade tasks.

Pupillary responses, particularly dilation in reaction to emotional stimuli, may also be modulated by GABAergic signaling. Dysregulation of GABA transmission in the LC could disrupt the coordination of pupillary dynamics with cognitive and emotional processing (Breton-Provencher and Sur, 2019). In rat models, activation of GABA_A_Rs has been shown to increase noradrenaline release from the LC via a facilitatory effect on glutamatergic terminals projecting to this region. Interestingly, this effect can be replicated by blocking NKCC1 with bumetanide, highlighting the role of GABAergic signaling and the NKCC1/KCC2 chloride balance in fine-tuning LC activity and, consequently, its influence on the oculomotor system (Koga et al., 2005; Capsoni et al., 2022). Furthermore, GABA levels in the visual cortex are linked to eye dominance and may modulate binocular visual competition, thereby affecting oculomotor control. Variations in GABAergic inhibition between the eyes during visual stimulation influence perceptual dominance and binocular rivalry dynamics, with pharmacological manipulation of GABA affecting perceptual switches and suppression (Ip et al., 2020). These findings collectively underscore the central role of GABAergic neurotransmission in the control of eye movements. Abnormal GABA neurotransmission can lead to measurable changes in oculomotor parameters. Overall, abnormalities in GABA synaptic transmission can lead to impaired performance in cognitive oculomotor tasks by affecting inhibitory control, sensory integration, and the synchronization of neural activity required for precise eye movements (Figure 8).

#### 2.6.1 Measure of oculomotor parameters during saccades, fixations, smooth pursuit

During the progression of AD, GABAergic dysfunction plays a critical role in the emergence of oculomotor deficits, reflecting the broader E/I imbalance characteristic of AD pathology (Bi et al., 2020). These deficits manifest across multiple domains of oculomotor control, including saccades, smooth pursuit, fixation, and inhibitory mechanisms. Patients with AD exhibit increased saccade latencies, hypometric saccades, and a higher frequency of saccadic intrusions during fixation. Additionally, they show impaired smooth pursuit eye movements, difficulty suppressing saccades during pursuit, and compromised fixation stability due to an inability to suppress reflexive saccades in antisaccade tasks (Molitor et al., 2015; Readman et al., 2021). These impairments provide insights into the broader cognitive and neural network disruptions in AD, highlighting the intricate relationship between neurotransmitter imbalances and motor outputs (Schmidt-Wilcke et al., 2018; Mattson, 2020; Xu et al., 2020; Maestú et al., 2021) (Figure 8).

Among these oculomotor markers, memory-guided saccades stand out as potential biomarkers for AD, given their reliance on spatial working memory and executive function. In AD, memory-guided saccades are characterized by decreased accuracy, prolonged latency, and reduced peak velocity, reflecting disruptions in neural circuits underlying spatial working memory (Pierrot-Deseilligny et al., 1991; 2002). The severity of cognitive decline correlates with the degree of neuronal network dysregulation, emphasizing the close link between synaptic dysfunction and cognitive performance. These impairments may stem from reduced GABAergic inhibition in the frontal and parietal cortices, areas crucial for working memory and saccade generation (Gao et al., 2024). Furthermore, the percentage of correct memory-guided saccades strongly correlates with verbal memory performance, particularly with Total Free and Delayed Recall tests in AD patients (Lage et al., 2021). This relationship suggests that GABAergic dysfunction impacts not only motor control but also fundamental memory processes (Williams et al., 2023). Additionally, memory-guided saccade impairments may aid in differentiating AD from other neurodegenerative disorders, such as semantic variant primary progressive aphasia, which exhibits distinct GABAergic dysfunction patterns (Lage et al., 2021).

In Posterior Cortical Atrophy (PCA) (Box 1), saccadic performance serves as a key diagnostic marker, with patients exhibiting prolonged fixation times, increased first major saccade latency, and significantly reduced saccade amplitudes (Shakespeare et al., 2015; Pin et al., 2023). These abnormalities reflect cognitive-perceptual processing deficits rather than mere motor dysfunction. Fixation stability is another major area of impairment, characterized by frequent large saccadic intrusions, shorter sustained fixations, and disrupted visual stabilization mechanisms (Shakespeare et al., 2015). These findings suggest a fundamental disruption in neural circuits responsible for visual attention and spatial orientation, possibly linked to alterations in GABAergic inhibition. Given its role in modulating visual perception and attention, disruptions in parietal and occipital cortical GABA levels could contribute to the observed deficits in spatial orientation and visual processing (Song et al., 2017; Gao et al., 2024; Kujala et al., 2024). Pursuit tracking further highlights the complexity of PCA, as patients exhibit diminished pursuit gain, increased saccadic intrusions, and reduced tracking precision (Shakespeare et al., 2015). These findings point to dysfunction in neural circuits responsible for smooth visual tracking and spatial integration, likely influenced by imbalances between GABAergic inhibition and glutamatergic excitation. Disruptions in GABAergic fine-tuning of neuronal responses in visual areas could lead to compensatory reliance on saccadic movements, impairing smooth pursuit (Thier and Ilg, 2005). Thus, the oculomotor profile in PCA goes beyond a mere symptomatic description, serving as a refined neurophysiological marker of posterior cortical network degeneration. The interplay between GABAergic dysfunction and visual processing deficits illustrates PCA’s complex pathophysiology and emphasizes the role of neurotransmitter imbalance in impairments of visual attention and tracking. Such vulnerabilities may trace back to early neurodevelopmental disturbances in magnocellular pathways, where initial E/I imbalance sets the stage for later network fragility. In this context, PCA exemplifies how network-specific susceptibilities rooted in developmental imbalance can manifest decades later as selective neurodegenerative syndromes, reinforcing the idea that oculomotor alterations may act as intermediate phenotypes and candidate biomarkers for preclinical AD.

#### 2.6.2 Measures of pupillary light reflex (PLR)

Early retinal changes have been reported in AD, with retinal abnormalities in the early stages including a specific pattern of retinal nerve fiber layer loss, narrowed veins, and decreased retinal blood flow. Amyloid deposits in the retina have been found at significantly higher levels in individuals with MCI, with a fivefold increase, and in those with AD, with a ninefold increase. These deposits may affect various retinal cells, particularly intrinsically photosensitive retinal ganglion cells (ipRGCs), especially melanopsin-expressing RGCs, which could, in turn, alter the pupillary light reflex (PLR) (Koronyo et al., 2017; 2023; Mirzaei et al., 2020). GABAergic transmission plays a crucial role in retinal ganglion cell function and visual processing. Some RGCs are themselves GABAergic, projecting to various brain regions, including the superior colliculus, where they contribute to transmitting looming signals and regulating innate defensive responses (Popova, 2015; Yuan et al., 2024). Additionally, ipRGCs express primarily GABA_A_- and GABA_C_Rs, which mediate inhibitory inputs that shape visual processing.

Most studies attribute GABA_c_R function to presynaptic modulation at bipolar cell synapses, rather than to direct postsynaptic effects on ipRGCs (Fletcher and Wässle, 1999; Zhu et al., 2007; Medina Arellano et al., 2025). This modulation shapes bipolar output and indirectly influences ipRGC responses, including pupillary and circadian rhythms. Although the direct involvement of GABA_C_​Rs in AD–related retinal or ocular pathology remains unclear, the loss of GABA_C_​R-mediated fine-tuning of retinal signaling may contribute to altered pupillary motility. Further studies are needed to clarify the contribution of retinal GABAergic mechanisms, particularly GABA_C_​R transmission, to AD-associated ocular changes.

Recent findings utilizing chromatic pupillometry have provided valuable insights into PLR abnormalities in AD. Findings suggest that detectable changes in the PLR emerge even in the early stages of the disease, potentially reflecting underlying pathological alterations in the dendritic processes of melanopsin-containing RGCs before the degeneration of their cell bodies (Romagnoli et al., 2020; 2023). While cholinergic neurotransmission has been widely recognized for its role in supporting the PLR, GABAergic regulation of the Edinger-Westphal nucleus and its potential alterations in AD may also play a significant role. Investigating the PLR across different stages of AD could offer further insights into the interplay between neurotransmitter dysfunction and disease progression (Chougule et al., 2019).

#### 2.6.3 Study of visual attention processing using cognitive task

##### 2.6.3.1 Reading task

Patients with AD typically exhibit distinctive eye movement patterns during reading tasks, characterized by reduced saccade amplitudes, prolonged fixation durations, higher regression rates, and more frequent and extended backward eye movements (Fernández et al., 2022). These oculomotor abnormalities provide valuable insight into underlying cognitive processing difficulties, particularly in phonological working memory and semantic integration. Dysfunction in GABAergic transmission may play a key role in these reading deficits, as GABA is essential for saccade control, visual cortex function, and overall oculomotor regulation, all of which are critical for fluent reading. Impaired GABAergic transmission can lead to increased saccadic frequency, more frequent fixations and regressions, prolonged fixation durations, and reduced saccade amplitudes, ultimately diminishing reading efficiency (Leventhal et al., 2003; Ibbotson and Krekelberg, 2011; Vidyasagar, 2019). Additionally, deficits in memory-guided saccades may further disrupt smooth text navigation, compounding difficulties in reading speed and comprehension. Specialized reading protocols integrating eye-tracking technology could help address these impairments by examining how AD patients process complex linguistic information, integrate semantic context, and manage cognitive load during language comprehension (Walenski et al., 2021). Analyses of fixation patterns, saccadic dynamics, and reading strategies provide a window into the mechanisms of progressive aphasia associated with neurodegenerative conditions. Beyond their clinical utility, such protocols may also illuminate AD pathophysiology by revealing parallels between abnormalities observed in neurodevelopmental disorders such as dyslexia and those in AD, particularly with respect to network vulnerabilities shaped by early E/I imbalance.

##### 2.6.3.2 Memory and other cognitive tasks

The current challenge in neurodegenerative disease research lies in integrating cognitive neuroscience data with oculomotor behavior analysis, particularly in the early stages of these conditions, with a focus on the role of GABAergic transmission impairments. GABAergic dysfunction is a key contributor to early memory impairment and may subsequently alter oculomotor behavior, particularly in AD. One manifestation of this dysfunction is hippocampal hyperexcitability due to reduced GABAergic transmission, leading to an E/I balance disruption that promotes epileptogenesis and memory deficits (Mao et al., 2024). Additionally, alterations in GABAergic signaling disrupt neural network synchronization and the regulation of neuronal activity in crucial areas such as the hippocampus, which plays an essential role in episodic memory (Hernández-Frausto et al., 2023). Furthermore, by modulating the balance between tonic and phasic firing modes, GABAergic transmission in the LC fine-tunes memory processes, optimizing cognitive performance across different behavioral contexts (Jin et al., 2016; Breton-Provencher and Sur, 2019). Several studies have demonstrated the potential of eye movement analysis as a tool for the early detection and prediction of cognitive decline (Zola et al., 2013)) found that the proportion of eye fixations in a visual pair memory task effectively predicted the conversion rate from MCI to major cognitive disorder over a 3-year period. Other research has identified differences in pupil dilation speeds during visual associative memory tasks between cognitively normal elderly individuals and those at risk of developing cognitive disorders (Dragan et al., 2017; Granholm et al., 2017) expanded on this by analyzing pupillary responses during working memory tasks, showing variations linked to the presence or absence of cognitive disorders and individual cognitive reserve. Notably, these ocular abnormalities were detectable even in seemingly normal individuals with a genetic predisposition to AD, as indicated by high polygenic risk scores (Kremen et al., 2019). Recent studies on healthy subjects have further evaluated the potential of pupillary response measurements in visual recognition memory. Findings indicate that pupillary dilation can distinguish between familiar and novel stimuli, with a stronger dilation response for previously encountered stimuli and a differentiation between familiarity and recollection-based memory processes (Otero et al., 2011; Kafkas and Montaldi, 2012; Gomes et al., 2015; 2021). These two processes engage distinct memory systems, particularly in terms of visual attention allocation. In AD, at the stage of amnestic MCI, early deficits in familiarity-based visual recognition memory could be detected through pupillary response differences. These variations may reflect individual performance levels, and the cognitive processes engaged during memory tasks, providing a potential biomarker for early disease detection and progression monitoring.

The precise measurement of oculomotor parameters offers a non-invasive approach to tracking the neurophysiological progression of AD, providing promising diagnostic and monitoring opportunities. By analyzing subtle changes in eye movement control, which reflect underlying GABAergic deficits, researchers and clinicians may be able to detect neurological alterations before pronounced cognitive symptoms emerge. Early detection could enable timely intervention and improve patient outcomes, particularly as ongoing research explores GABAergic therapies for AD. Integrating oculomotor assessments with other biomarkers may lead to more comprehensive and sensitive diagnostic strategies, ultimately enhancing patient care and treatment approaches.

Tailored experimental protocols could be designed to accommodate the specific eye movement patterns and cognitive challenges observed in AD patients, potentially improving reading performance and overall language processing. The convergence of eye movement analysis and cognitive neuroscience underscores its potential as a non-invasive early indicator of neurodegenerative processes. Understanding the relationship between pathological chloride homeostasis and oculomotor characteristics provides crucial insights into underlying cognitive processing deficits, particularly in phonological working memory, semantic integration, and broader memory functions. This knowledge highlights the intricate interplay between neurotransmitter systems, cognitive function, and motor control. Such insights may pave the way for novel therapeutic interventions targeting the GABAergic system via cation-chloride co-transporters (Capsoni et al., 2022; Lam et al., 2022; Kadam and Hegarty, 2024), aiming to improve both cognitive function and oculomotor control in neurodegenerative diseases. Restoring chloride homeostasis could offer a novel therapeutic strategy to alleviate cognitive and motor symptoms in AD, potentially improving patients’ quality of life and preserving functional independence.

The intricate relationship between synaptic dysfunction, neural network disruption, and oculomotor performance in AD highlights the need for comprehensive, multidimensional approaches to both understanding and developing potential interventions for this neurodegenerative condition.

### 2.7 Therapeutic perspectives: targeting chloride transport and GABAergic dysfunction

Disrupted chloride homeostasis has emerged as a central feature of AD, with growing evidence implicating the dysregulation of chloride co-transporters, particularly KCC2 and NKCC1, in early E/I imbalances, cortical hyperexcitability, and cognitive decline. These alterations compromise the polarity of GABAergic signaling, leading to reduced inhibition or even paradoxical excitation, which contributes to abnormal network synchronization and neurodegeneration. Therapeutic strategies aimed at restoring GABAergic inhibition by modulating chloride transport hold significant promise. Enhancing KCC2 function or inhibiting NKCC1 has shown beneficial effects in various preclinical models of brain disorders, including epilepsy, traumatic brain injury, and AD (Capsoni et al., 2022; Hochstetler et al., 2025). Although bumetanide, a known NKCC1 inhibitor, has demonstrated neuroprotective effects, its clinical utility in AD remains limited due to poor brain penetration and systemic side effects (Glykys et al., 2014; Töllner et al., 2014).

Beyond direct pharmacological modulation, several experimental approaches aim to indirectly stabilize KCC2. These include reducing neuroinflammation, mitigating oxidative stress, or enhancing neurotrophic support via BDNF-TrkB signaling. For instance, activation of TrkB receptors or improving proBDNF-to-BDNF maturation promotes KCC2 expression and functional recovery (Rivera et al., 2004; Tang et al., 2019). Similarly, exosome-based therapies derived from adipose mesenchymal stem cells have shown efficacy in modulating inflammatory cascades and preserving chloride balance (Li P. et al., 2024). Inhibition of the NF-κB/NLRP3 pathway may also rescue KCC2 expression and reduce synaptic damage (Bergauer et al., 2022).

The therapeutic relevance of KCC2 has expanded with recognition of its dual function, not only in regulating intracellular chloride, but also in stabilizing dendritic spines and supporting synaptic plasticity (Gauvain et al., 2011; Virtanen et al., 2021). In consequence, loss of KCC2 thus affects both neuronal signaling and network architecture. Recent studies have highlighted post-translational mechanisms, such as phosphorylation and membrane trafficking, as promising targets for more specific therapeutic interventions (Friedel et al., 2015; Tang et al., 2019; Hartmann and Nothwang, 2022). Encouragingly, strategies aimed at restoring KCC2 function have shown considerable promise. For instance, pharmacological enhancement of KCC2 activity using small molecules like CLP290 reinstates inhibitory neurotransmission, normalizes chloride homeostasis, and reverses cognitive deficits in AD mouse models (Keramidis et al., 2023).

In parallel, emerging approaches are focusing on modulating network oscillations, particularly gamma oscillations, which are closely tied to GABAergic interneuron function and cognitive performance. Abnormal gamma activity is an early hallmark of AD and correlates with synaptic dysfunction and memory impairment. Restoration of gamma oscillations is thus a key therapeutic objective, aiming to counteract pathological hyperexcitability, reduce amyloid and tau pathology, normalize microglial activity, and improve cognitive outcomes.

Neuromodulation strategies have demonstrated promising effects in preclinical models. For instance, optogenetic stimulation of PV interneurons at 40 Hz reduces amyloid plaque burden by enhancing frequency-specific gamma-oscillations and improving network synchrony (Iaccarino et al., 2016). Although highly informative mechanistically, optogenetics faces major hurdles for clinical application due to its invasiveness. Non-invasive alternatives, including sensory stimulation (visual or auditory 40 Hz entrainment) and brain stimulation techniques such as transcranial magnetic stimulation and transcranial alternating current stimulation, offer more translational potential (Toniolo et al., 2020). However, challenges such as timing mismatches with endogenous oscillations and variable clinical efficacy highlight the need for more reliable interventions.

Pharmacological strategies provide a compelling alternative by directly targeting the molecular underpinnings of gamma-oscillation deficits. Recent efforts have focused on modulating GABAergic inhibition in PV interneurons, particularly in the context of KCC2 dysfunction, which disrupts chloride homeostasis and impairs inhibitory tone (Wei et al., 2024). A notable example is DDL-920, a negative allosteric modulator of extrasynaptic α1β2δ GABA_A_Rs. By selectively reducing excessive tonic inhibition (achieving a 71% suppression at 1 nM) while sparing phasic transmission, DDL-920 restores PV interneurons excitability and enhances gamma-power (2.5-fold increase), effectively bypassing the need for direct KCC2 reactivation. This pharmacological approach addresses key limitations of neuromodulation by providing sustained, cell-type-specific modulation of gamma-oscillations independent of external stimuli.

Future therapeutic development may benefit from combination strategies that integrate sensory entrainment with chloride-targeted therapies or GABAergic modulators like DDL-920, aiming for a more robust and durable restoration of network function. Such approaches are particularly attractive given their low invasiveness and potential for synergistic effects. By promoting synchronous GABAergic activity, gamma entrainment may indirectly support KCC2 function, reinforcing inhibitory tone and enhancing cognitive performance. The transition from optogenetic proof-of-concept studies to clinically viable pharmacological agents mark a significant advance toward precision medicine for gamma-oscillation deficits in neurodegeneration.

In addition, innovative tools such as eye-tracking in transgenic models with KCC2 alterations (Ambrad Giovannetti and Rancz, 2024) are emerging as non-invasive biomarkers of GABAergic function and network integrity. These platforms hold great promise for monitoring therapeutic efficacy and network-level responses in both preclinical and clinical settings.

Despite encouraging progress, several gaps remain. The timeline of chloride dysregulation during AD progression is not well defined, and validation in human postmortem tissue is still limited (Zhou et al., 2021; Kreis et al., 2023). Moreover, the interplay between KCC2 and other chloride transporters, such as NKCC1, requires further investigation (Lam et al., 2022). Nevertheless, targeting chloride homeostasis, particularly in combination with neuromodulatory strategies, represents a sophisticated and increasingly promising avenue for restoring inhibitory balance and mitigating neurodegeneration in AD.

## 3 General conclusion and future directions

**FIGURE 9:**
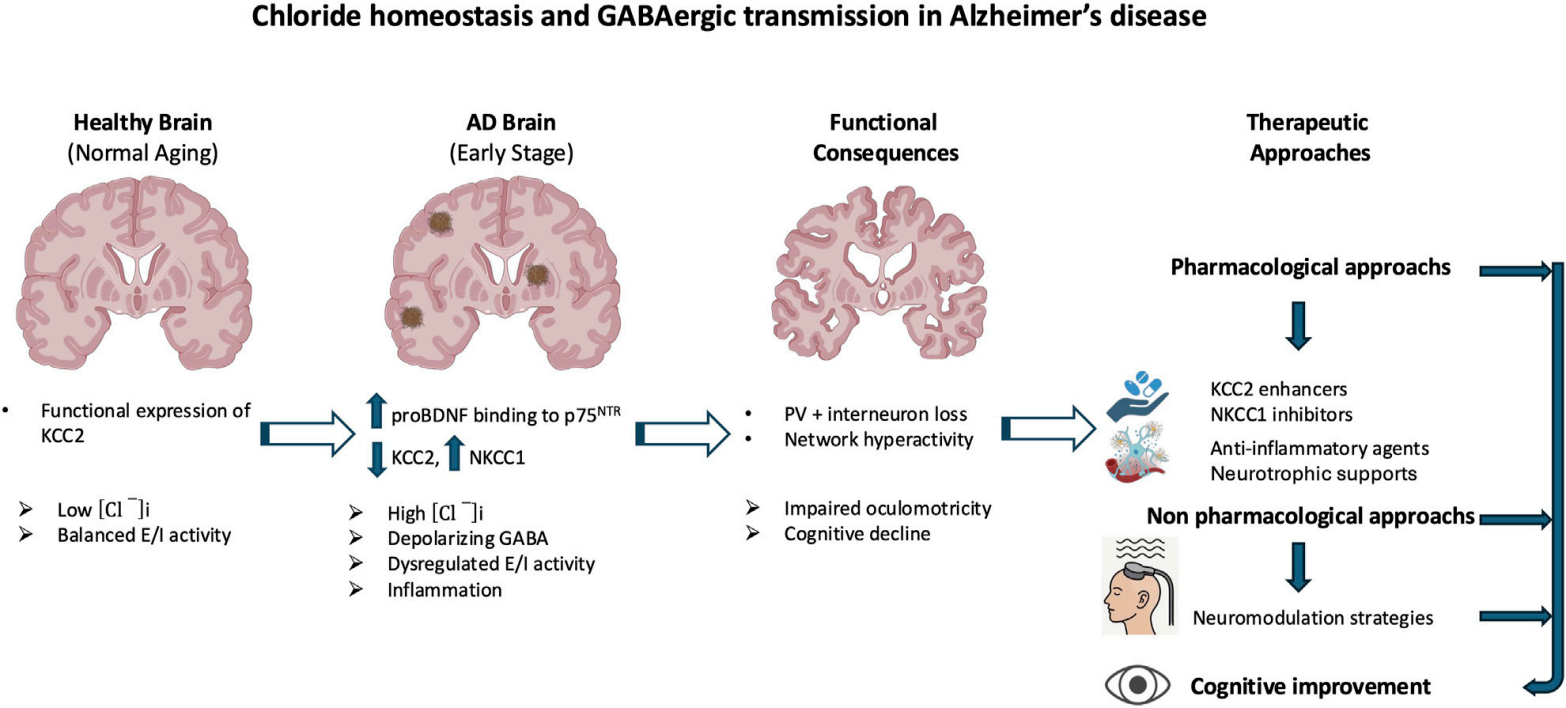
Disruption of chloride homeostasis and GABAergic dysfunction in Alzheimer’s disease (AD). The diagram illustrates how early downregulation of KCC2 and upregulation of NKCC1 in AD impair GABAergic inhibition and disrupt chloride homeostasis. These changes contribute to cortical network hyperexcitability, loss of parvalbumin-positive (PV) interneurons, and altered gamma oscillations. These pathophysiological processes are further exacerbated by neuroinflammation, amyloid-β toxicity, and deficits in BDNF- TrkB signaling, ultimately leading to oculomotor dysfunction and cognitive deficits. Emerging therapeutic strategies aim to restore inhibitory balance through pharmacological modulation of chloride transporters, enhancement of neurotrophic support, and anti-inflammatory interventions in combination with neuromodulatory strategies. KCC2 has become a central therapeutic target due to its dual role in ion regulation and synaptic structure maintenance.

GABAergic dysfunction, driven largely by disrupted chloride homeostasis, emerges as a key early event in the pathogenesis of AD. The downregulation of KCC2 and upregulation of NKCC1 compromise inhibitory signaling, disturb E/I balance, and contribute to cortical hyperexcitability, impaired gamma oscillations, and cognitive decline. These alterations are further exacerbated by neuroinflammation, Aβ toxicity, and deficits in neurotrophic signaling, particularly involving BDNF. Understanding the interplay between gamma oscillations, E/I balance, and GABAergic inhibition is thus essential for the development of targeted therapies in AD. Emerging approaches, including pharmacological modulation of chloride transporters, enhancement of BDNF-TrkB signaling, and anti-inflammatory interventions, seek to correct inhibitory deficits at multiple levels. KCC2 has become a central therapeutic target due to its dual role in regulating ion gradients and maintaining synaptic structure (Figure 9).

Until now, translation to clinical application remains challenging. Many candidate therapies are still at the preclinical stage, and the heterogeneity of AD pathology, combined with barriers to brain delivery and uncertainties about therapeutic timing, complicate the development of broadly effective interventions. Longitudinal studies, improved biomarkers, and refined animal models will be critical for determining when and how to intervene most effectively.

Altogether, these findings suggest that restoring chloride homeostasis to rebalance inhibitory signaling represents a promising avenue for disease modification in AD. As our understanding of the molecular and circuit-level underpinnings of GABAergic dysfunction deepens, so too does the potential for personalized, mechanism-based treatments aimed at halting or even reversing cognitive decline in neurodegenerative disease.

## Abbreviations

AD, Alzheimer’s disease; Aβ, Amyloid-beta; BDNF, Brain-derived neurotrophic factor; dlPFC, Dorsolateral prefrontal cortex; EWN, Edinger-Westphal nucleus; E/I, Excitatory/inhibitory; FEF, Frontal eye fields; FOF, Frontal oriented field; IL, Infralimbic, prelimbic (PL) cortices; ipRGCs, Intrinsically photosensitive retinal ganglion cells; LC, Locus coeruleus; mPFC, Medial prefrontal cortex; MCI, Mild cognitive impairment; PV, Parvalbumin-positive interneurons; PCA, Posterior cortical atrophy; KCC2, Potassium-chloride co-transporter 2; PLR, Pupillary light reflex; RGCs, Retinal ganglion cells; NKCC1, Sodium-potassium-chloride co-transporter; SNr, Substantia nigra pars reticulata; SC, Superior colliculus; TBI, Traumatic brain injury.
